# Ratiometric imaging of minor groove binders in mammalian cells using Raman microscopy†

**DOI:** 10.1039/d2cb00159d

**Published:** 2022-09-26

**Authors:** Christian Tentellino, William J. Tipping, Leah M. C. McGee, Laura M. Bain, Corinna Wetherill, Stacey Laing, Izaak Tyson-Hirst, Colin J. Suckling, Rebecca Beveridge, Fraser J. Scott, Karen Faulds, Duncan Graham

**Affiliations:** Centre for Molecular Nanometrology, WestCHEM, Department of Pure and Applied Chemistry, Technology and Innovation Centre, University of Strathclyde Glasgow G1 1RD UK karen.faulds@strath.ac.uk duncan.graham@strath.ac.uk; Department of Pure and Applied Chemistry, Thomas Graham Building, University of Strathclyde Glasgow G1 1XL UK fraser.scott@strath.ac.uk

## Abstract

Quantitative drug imaging in live cells is a major challenge in drug discovery and development. Many drug screening techniques are performed in solution, and therefore do not consider the impact of the complex cellular environment in their result. As such, important features of drug–cell interactions may be overlooked. In this study, Raman microscopy is used as a powerful technique for semi-quantitative imaging of Strathclyde-minor groove binders (S-MGBs) in mammalian cells under biocompatible imaging conditions. Raman imaging determined the influence of the tail group of two novel minor groove binders (S-MGB-528 and S-MGB-529) in mammalian cell models. These novel S-MGBs contained alkyne moieties which enabled analysis in the cell-silent region of the Raman spectrum. The intracellular uptake concentration, distribution and mechanism were evaluated as a function of the p*K*_a_ of the tail group, morpholine and amidine, for S-MGB-528 and S-MGB-529, respectively. Although S-MGB-529 had a higher binding affinity to the minor groove of DNA in solution-phase measurements, the Raman imaging data indicated that S-MGB-528 showed a greater degree of intracellular accumulation. Furthermore, using high resolution stimulated Raman scattering (SRS) microscopy, the initial localisation of S-MGB-528 was shown to be in the nucleus before accumulation in the lysosome, which was demonstrated using a multimodal imaging approach. This study highlights the potential of Raman spectroscopy for semi-quantitative drug imaging studies and highlights the importance of imaging techniques to investigate drug–cell interactions, to better inform the drug design process.

## Abstract 2

The uptake and localisation of minor groove binders into mammalian cells is visualised using alkyne-tag Raman imaging.

## Graphical abstract

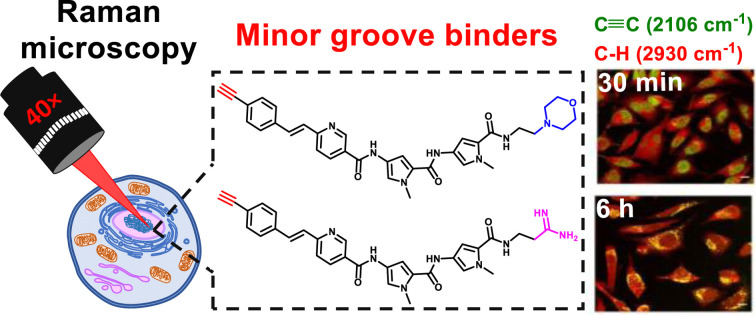

## Introduction

Minor groove binders (MGBs) are a class of compounds that interact with the minor groove of DNA. Heterocyclic polyamides, aryl benzimidazoles, aryl amidines, pyrrolobenzodiazepines, isoquinolines and benzofurans have been developed to target the minor groove through a variety of means including ionic bonding, hydrophobic interactions and hydrogen bonding.^1^ Such compounds have found utility in drug discovery as therapeutic leads against multiple pathogens including bacteria, fungi, viruses, parasites and yeasts and also in cancer therapy.^2–5^ The potential of MGBs as development candidates is often questioned due to healthy mammalian cells also possessing a similar genomic DNA target, suggesting potential cytotoxicity problems. However, in many cases excellent selectivity indices can be achieved, with several MGBs used in the clinic or in clinical trials.^5^ Not only is DNA binding significant in the action of MGBs, intracellular uptake and distribution also play important roles.^6^ Reduced intracellular accumulation is also a known mechanism of resistance to some antiparasitic MGBs.^7^ The diamidines, including pentamidine, propamidine and stilbamidine, enter trypanosomes *via* the P2 transporter, and either loss or down-regulation of this transporter results in pathogen resistance which has been frequently observed. Despite the recent success of MGB drugs in clinical practice against translocation-associated sarcomas (trabectedin)^8^ and metastatic lung cancer (lurbinectedin),^9^ only limited progress has been made in assessing the uptake, distribution and activity of MGBs in mammalian cells in preclinical settings due to a lack of appropriate imaging tools to study MGB uptake and distribution in a biological model.

Advanced imaging techniques have been employed in the early stages of drug discovery to identify novel aspects of drug activity at the cellular level.^10^ Of these techniques, mass spectrometry is destructive and offers a relatively low resolution (typically 20 μm),^11^ whilst fluorescence microscopy, which has been most extensively applied in this area, is hampered by the requirement of bulky fluorophores (>300 Da) which, in many cases, are larger than the drug molecule they are conjugated to.^12^ As such, the fluorescent label may lead to incorrect conclusions in studies concerning drug uptake and localisation. This issue was exemplified by Dervan *et al.* who identified that a fluorophore contributed to the redistribution of BODIPY-conjugated polyamides following chemical fixation prior to cellular imaging.^13^ Some MGBs are inherently fluorescent, as is the case for DAPI and Hoechst dyes.^14^ However, relying on inherent fluorescence of lead compounds greatly restricts the diversity in chemical motifs that can be imaged in this way. Label-free or minimally perturbative labelling strategies thus offer a preferential methodology to study MGBs in live cells.

Raman spectroscopy can provide direct visualisation of cellular biomolecules in a label-free or minimally perturbative manner.^15^ Xie *et al.* demonstrated the detection of two tyrosine kinase inhibitors, imatinib and nilotinib, in chronic myeloid leukaemia (CML) cells using hyperspectral stimulated Raman scattering (SRS) microscopy.^16^ These drugs were shown to accumulate more than 1000-fold in lysosomes of CML cells. To enhance the detection of drug molecules, small bio-orthogonal groups including, alkynes and nitriles have been incorporated into drug scaffolds *via* tagging,^15^ or may even be present in the parent molecule itself *e.g.* ponatinib^17^ and neratinib,^18^ to enable detection using Raman scattering techniques. Alkynes and nitriles generate peaks in the cell-silent region of the Raman spectrum (1800–2600 cm^−1^) and therefore offer specificity from the endogenous cellular signals. MGBs have yet to be studied using bio-orthogonal Raman imaging, which offers the potential for studying the mechanism of uptake and action in a minimally perturbative manner.

**Fig. 1:**
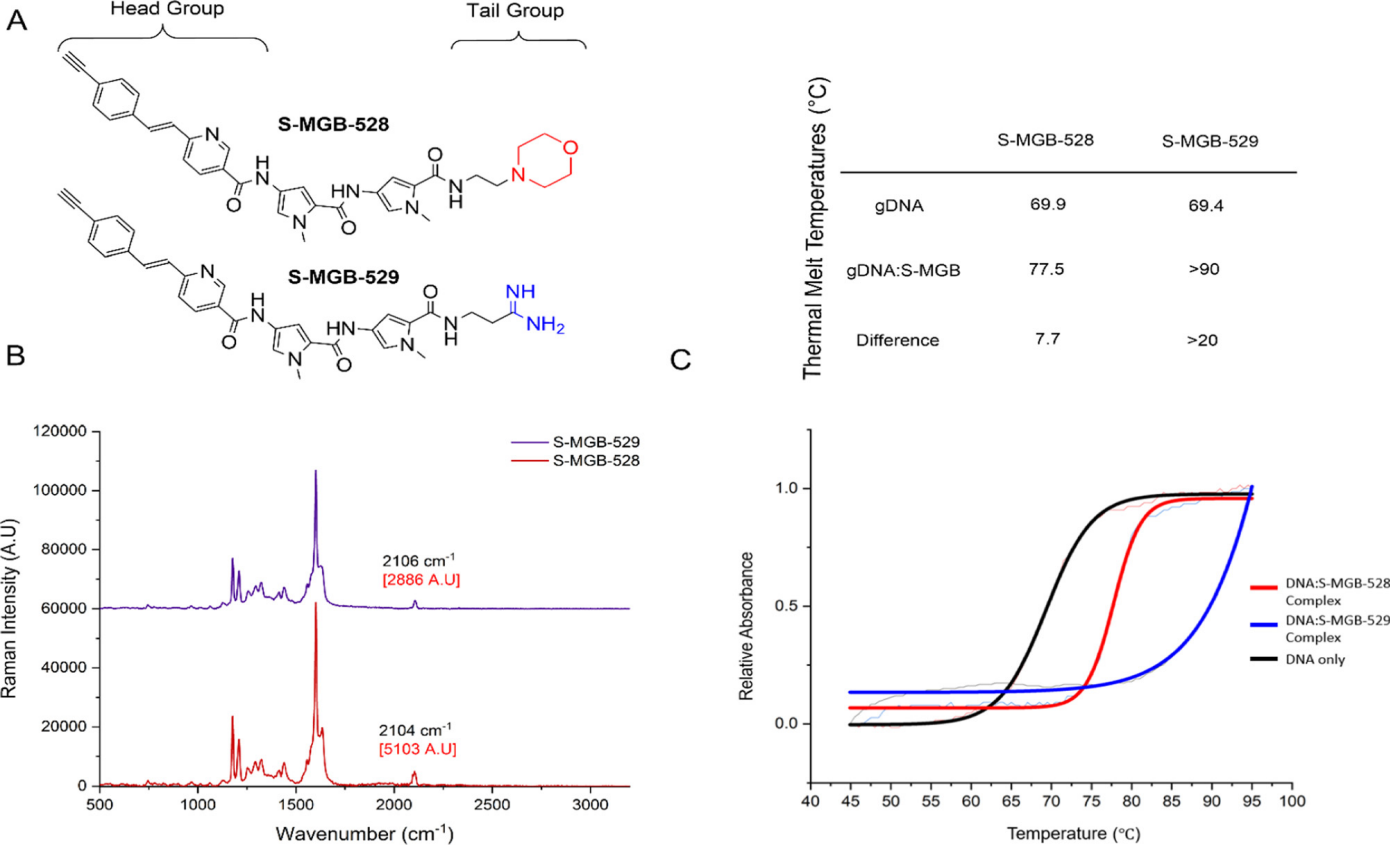
Analysis of S-MGBs using Raman spectroscopy. (A) General design and chemical structures of truncated S-MGBs, S-MGB-528 (morpholine tail group) and S-MGB-529 (amidine tail group). (B) The Raman scattering of the alkyne-tagged S-MGBs in a solid state. (C) Thermal melt analysis of S-MGB-528 or S-MGB-529 bound to gDNA. Exemplar melt curve from one experimental repeat, which has been fitted with a Boltzmann distribution and inset table including average thermal melts from at least 2 experimental repeats. The errors of the average thermal melts are within ±0.5 °C.

Herein, we present the first example of a Raman imaging-based approach to study the uptake, distribution and cytotoxicity of MGBs in live mammalian cells, specifically, Strathclyde-Minor Groove Binders (S-MGBs), based on the structure of the natural product distamycin, which have been shown to have potent anti-infective activities.^19–23^ One such S-MGB is MGB-BP-3, which has successfully completed a phase IIa clinical trial for the treatment of *Clostridioides difficile* infection, and others have demonstrated *in vivo* activity in proof-of-concept studies against parasitic, fungal and viral pathogens.^19–23^ Structure-activity relationships have recently established a crucial role for the ‘tail group’ moiety of S-MGBs (Fig. 1A) in modulating activity profiles against different pathogens.^24,25^ It is emerging that S-MGBs with higher p*K*_a_ amidine ‘tail groups’ are consistently less cytotoxic than those with lower p*K*_a_ morpholine ‘tail groups’ (unpublished results); however, the underlying biological mechanism governing this difference in cytotoxicity is not understood. Therefore, this study aims to investigate the intracellular uptake and distribution of S-MGBs at the subcellular level in order to explain the different biological consequences of ‘morpholine’ and ‘amidine’ tail groups and exemplify where the power of SRS microscopy can be utilised.

## Results and discussion

### S-MGB-528 and S-MGB-529 behave like other S-MGBs

We employed an alkyne-tag Raman imaging (ATRI)-based approach (where the alkyne used for labelling is <40 Da) to enable highly sensitive detection of the S-MGBs in the cell-silent region of the Raman spectrum (1800–2600 cm^−1^) expecting minimal impact on the biological activity of the compound.^15^ Two new S-MGBs bearing an alkyne tag, one with a morpholine ‘tail group’, S-MGB-528, and the other an amidine ‘tail group’, S-MGB-529, were synthesised using established methods (Fig. 1A and Schemes S1, S2, ESI†).

We first acquired a Raman spectrum from a solid sample of each S-MGB using NIR excitation (785 nm). Fig. 1B presents the Raman spectra of the S-MGBs in solid form; an intense alkyne peak is observed at 2104 cm^−1^ and 2106 cm^−1^ for S-MGB-528 and S-MGB-529, respectively. We next validated the minor groove binding of each compound using established methods. Firstly, thermal shift assays, using genomic DNA (salmon, Fig. 1C) and native mass spectrometry (Fig. S1 and S2, ESI†), using a short, self-complementary dsDNA oligomer (5′-GCGATATATCGC-3′), confirmed that both S-MGB-528 and S-MGB-529 bind to dsDNA, as expected (Fig. 1C and Fig. S1, S2, ESI†). Moreover, both S-MGBs bind to dsDNA as a dimer (Fig. S1 and S2, ESI†), with the amidine containing S-MGB-529 exhibiting a greater stabilisation of dsDNA (Fig. 1C, inset table), which is in line with other S-MGBs.

Since the efficacy and cytotoxicity of MGBs have been associated with target cellular uptake and accumulation, it is necessary to investigate the uptake and cytotoxicity of S-MGB-528 and S-MGB-529 in representative mammalian cell lines. We elected to study S-MGB-528 and S-MGB-529 in PNT2 cells as a model of a healthy mammalian cell line, whilst HeLa cells were used given their extensive previous use as a model cell line to investigate MGB uptake and DNA targeting.^14,26,27^ Our data indicated that in both PNT2 and HeLa cell lines, the amidine (S-MGB-529) had a lower cytotoxicity than the morpholine (S-MGB-528) (Fig. S3, ESI†). For example, when HeLa cells were treated with each compound at a concentration of 10 μM, the percentage of viable cells after 24 h was 27% and 50% for S-MGB-528 and S-MGB-529, respectively. In addition, our data indicated that HeLa cells showed a greater sensitivity to both drugs when compared to PNT2 cells, the reason for which remains unclear. These data are in agreement with previous cytotoxicity studies of morpholine *vs.* amidine S-MGBs.^28^ Collectively, these experiments confirm that S-MGB-528 and S-MGB-529 behave similarly to other S-MGBs in both cellular and molecular environments and are therefore suitable Raman probes for exploring the underlying biological mechanisms that may account for the different cytotoxicity profiles of morpholine and amidine containing S-MGBs.

### The intracellular accumulation of S-MGB-529 is lower than S-MGB-528, in line with its lower cytotoxicity

**Fig. 2:**
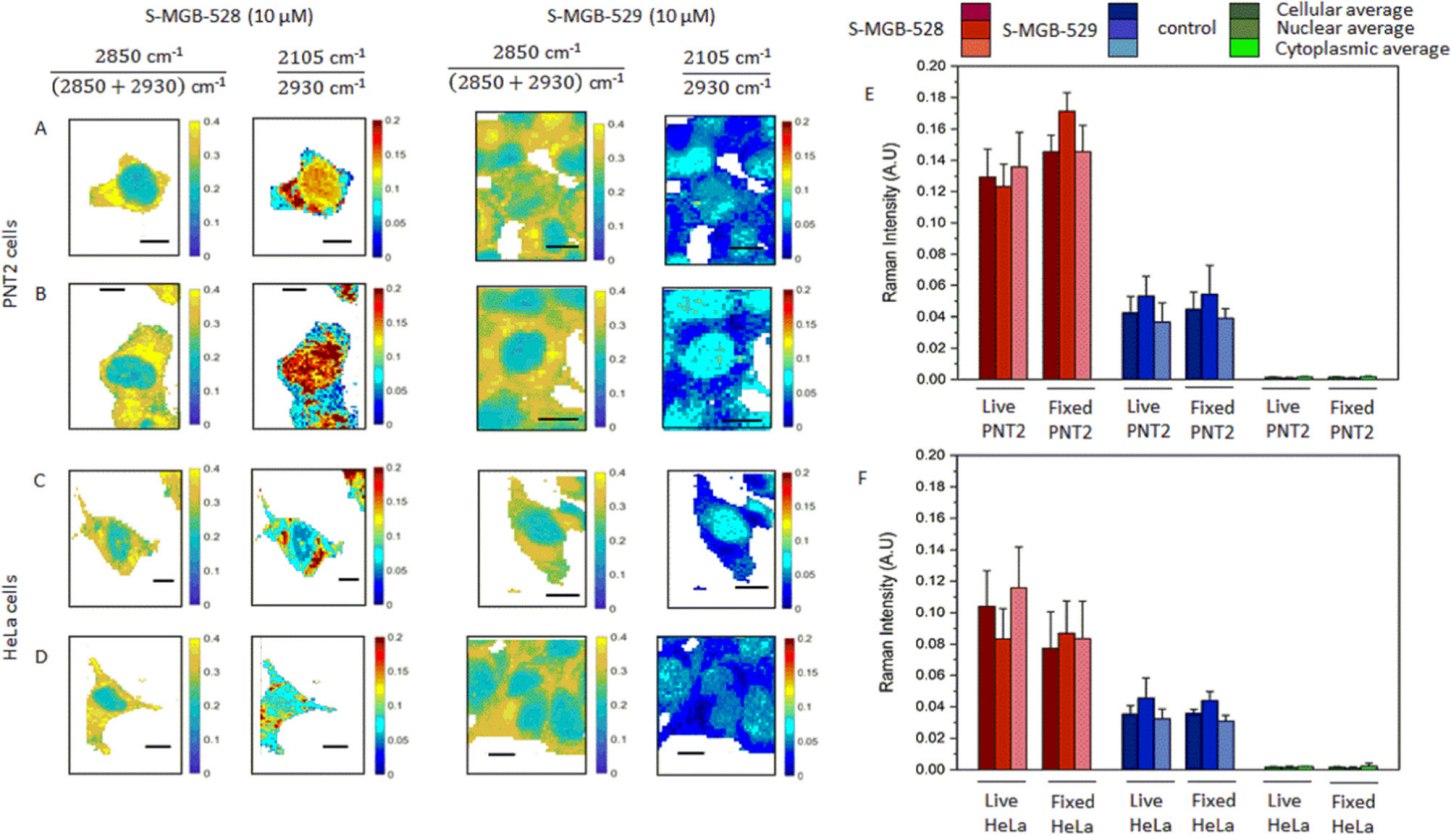
Ratiometric Raman analysis of S-MGBs in mammalian cells. Representative Raman maps of live (A and C) and fixed (B and D) PNT2 and HeLa cells after the treatment with either S-MGB-528 or S-MGB-529 (10 μM, 24 h, 37 °C, 5% CO_2_). The chemical contrast is shown by the ratio 2850 cm^−1^/(2850 + 2930) cm^−1^ indicative of CH_2_ and CH_3_ respectively. The drug localisation is shown through the normalised alkyne Raman intensity (*i.e.* 2105–2930 cm^−1^). Quantification of the normalised Raman scattering intensity of the alkyne tag (~2105 cm^−1^) relative to the symmetric C–H stretching (2930 cm^−1^) in live and fixed cells, (E) PNT2 and (F) HeLa cells, respectively. Raman data were collected using 532 nm, 10 mW laser power, 0.5 s integration time, 60× immersive objective lens, 1 μm step size in *x* and *y*, 1 accumulation. Data representative of the mean ± SD. A minimum of three replicate images were acquired for each condition.

Having established the suitability of the two Raman-labelled S-MGBs, we then investigated the uptake of S-MGB-528 and S-MGB-529 into live and fixed cells using ratiometric Raman spectroscopy.^29^ S-MGBs are usually effective against pathogens in the range between 50 and 1000 nM, with cytotoxicity against mammalian cells only at much higher concentrations.^20,30,31^ In this study, a higher S-MGB concentration was used to purposely investigate the intracellular uptake and distribution of S-MGBs in relation to cytotoxic effects. HeLa and PNT2 cells were treated with S-MGB-528 or S-MGB-529 at a concentration of 10 μM for 24 h, and Raman images acquired using 532 nm excitation, 10 mW laser power, 0.5 s integration time, 60× immersive objective lens, peak centre 2600 cm^−1^ and 1 μm step size in *x* and *y*, from both live and fixed (4% PFA in PBS) cells. Fig. 2 presents the ratiometric Raman maps of the intensity ratio 1 : 2850 cm^−1^/(2850 + 2930 cm^−1^) based on the CH_2_ and CH_3_ symmetric stretch attributed to lipids and proteins, respectively.^29^ This analysis readily resolves the nuclear (ratio 1 ≈ 0.2 a.u.) and cytoplasmic regions (typically ratio 1 > 0.25 a.u.) of the cells. We were then able to assess the distribution of S- MGB-528 and S-MGB-529 in the cellular populations based on the intensity of the alkyne signal relative to the CH_3_ (proteins) signal using the ratio: alkyne cm^−1^/2930 cm^−1^.

A threshold analysis was applied to the acquired Raman images in Fig. 2 to enable the semi-quantitative analysis of the uptake of both compounds within the nucleus, cytoplasm and across the cell as a whole in both cell populations. To do so, an intensity threshold was applied to ratiometric Raman imaging data sets (Fig. 2A–D) to separate the nuclear region (ratio 1 ≤ 0.24 a.u.) and the cytoplasm region (ratio 1 ≥ 0.25 a.u.) as above. From this, we calculated the average Raman spectrum of all the pixels within the nucleus and cytoplasm regions and compared these to the total cellular Raman spectrum (all pixels within the image). The mean intensity of the alkyne stretching frequencies are provided in Fig. 2E and F, whilst the Raman spectra are provided in Fig. S4A (ESI†). Both S-MGBs have a similar Raman scattering cross section in DMSO when the alkyne intensities were normalised to the Raman activity assigned to the DMSO (2910 cm^−1^) and therefore comparisons between the different S-MGBs can be made (Fig. S4B, ESI†). This analysis highlights that there is a greater uptake of S-MGB-528 compared to S-MGB-529 in both cell lines (2105/2930 cm^−1^). Additionally, S-MGB-528 displays a predominant cytoplasmic localisation in live PNT2 (Fig. 2A) and HeLa cells (Fig. 2C), with some nuclear localisation and the greatest uptake observed in PNT2 cells. In contrast, S-MGB-529 is mostly associated with the nuclear regions. Paraformaldehyde fixation did not result in an overall loss of the intracellular presence of the S-MGBs but in a partial redistribution of S-MGB-528 within the nucleus of PNT2 (Fig. 2A and B, *p* < 0.05) and HeLa (Fig. 2C and D, *p* ≥ 0.5) cells. The p*K*_a_ of the tail group of S-MGB-528 (p*K*_a_ = 6; morpholine) and S-MGB-529 (p*K*_a_ = 13; amidine) and the difference may go some way to rationalise this observation, with a potential alteration of the pH gradient between acidic organelles and cytosol being more likely responsible for the redistribution observed, with the p*K*_a_ of the tail moiety more likely involved in this effect.

A comparison of the behaviours of S-MGB-528 and S-MGB-529 suggests that there is an association between cytotoxicity and the uptake concentration of S-MGB (Fig. 2 and Fig. S3, ESI†); S-MGB-528 was more cytotoxic and had higher uptake in both cell lines, highlighting the importance of molecular structure and properties in drug uptake, distribution and cytotoxicity.

Closer inspection of the average Raman spectra from each cellular region are provided in Fig. S5 (ESI†) which show that the alkyne stretching frequency is red shifted in the average nuclear Raman spectrum compared to the average cytoplasmic Raman spectrum for both compounds in both cell lines. This result suggested that the terminal alkyne group of both molecules is sensitive to the local environment. To test this hypothesis, we used a model in which the peak profile of the alkyne tags of the S-MGBs was investigated in different aqueous/organic mixtures which replicate the hydrophobic environment of the minor groove compared to the overall nuclear region.^32^ We acquired Raman spectra of both S-MGBs in aqueous/organic mixtures (Fig. S5, ESI†). As the DMSO content of the mixture increased, a corresponding red shift of the alkyne stretching frequencies was observed (S-MGB-528: from 2103.0 to 2096.8 cm^−1^; S-MGB-529: from 2102.6 to 2098.4 cm^−1^). Considering that a red shift of the alkyne groups was observed in the average nuclear Raman spectra, compared to the average cytoplasmic spectra, this result suggests that drugs are bound to the minor groove, which is locally hydrophobic.^32^ Therefore, the terminal alkyne is extremely sensitive to the chemical composition of the surrounding milieu, and could enable ratiometric sensing of the binding of S-MGB-528 and S-MGB-529 to the minor groove.

We quantified the concentration of each S-MGB in the nucleus, cytoplasm and whole cell from the respective average Raman spectra based on calibration curves for each compound (Fig. S6, ESI†). These data are tabulated in Table S10 (ESI†) and show that the uptake of S-MGB-528 is >1 mM, representing >100-fold enrichment of the drug in the cell compared to the extracellular treatment concentration (10 μM). In the case of the amidine S-MGB-529, a lower cellular uptake was determined in both HeLa and PNT2 cells (*ca.* 0.4 mM), with an enrichment ratio of ≈40-fold. We then measured the intracellular concentrations of the S-MGBs in the nucleus, cytoplasm and the whole cell as a ratio (S-MGB-528/S-MGB-529) to investigate the different intracellular accumulation at the subcellular level. Interestingly, in PNT2 cells, this ratio remains consistently around 3 whilst in HeLa cells, this ratio varied considerably (nucleus = 5, cytoplasm = 14, cell = 10). This suggests that the subcellular distribution of the compounds depends on the specific cell type. Nonetheless, for both cell types investigated, the concentration of S-MGB-529 is consistently lower than that of S-MGB-528 in both nuclei and cytoplasm, in line with its lower cytotoxicity. The greater uptake of the morpholine S-MGB-528 in live cells compared to the amidine S-MGB-529 suggests that the tail group p*K*_a_ is significant in the intracellular accumulation. Specifically, at physiological pH (pH 7.4), the amidine S-MGB-529 is protonated (p*K*_a_ 13). As such, S-MGB-529 may be less cell penetrant than S-MGB-528, bearing the morpholino tail, if the uptake of this molecule is reliant upon passive diffusion only. We therefore proposed a Raman-based study in which we investigated the potential effect of the lipid membrane on the S-MGBs’ intracellular uptake and the uptake mechanism of each of these compounds.

### S-MGBs cannot freely pass the lipid membrane

A lower uptake and cytotoxicity of S-MGB-529 compared to S-MGB-528 was likely due to the charge of the amidine tail, rendering the molecule less able to pass through the cell membrane. To test this hypothesis, we investigated the uptake of S-MGBs in fixed cells (10 μM, 10 min) and compared this with the uptake into fixed and permeabilised cells (10 μM, 10 min) (Fig. S7, ESI†). Paraformaldehyde fixation has already been shown to affect the subcellular distribution of S-MGBs, although the overall intracellular concentration of the S-MGBs was not affected (Fig. 2). Permeabilization using Triton-X-100 (0.1%, 10 min), increases the porosity of the lipid membrane of fixed cells. Any difference between fixed cells and fixed, permeabilised cells would imply the involvement of the lipid membrane in S-MGB uptake due to a different integrity of the lipid membrane prior to the permeabilisation step (low porosity *vs.* high porosity). A greater uptake of both compounds was measured in fixed, permeabilised mammalian cells compared to cells that were only fixed (Fig. S7, ESI†). Again, PNT2 cells showed a higher S-MGB uptake than HeLa cells. This suggests that neither S-MGB-528 or S-MGB-529 are able to pass through the lipid membrane freely. Furthermore, both S-MGBs localised predominantly in the nucleus and the permeabilisation did not cause any further redistribution of the S-MGBs. As such, we suggest that the redistribution observed when PNT2 and HeLa cells were treated with S-MGB-528 for 24 hours (Fig. 2) was associated with the loss in the pH gradient of the lysosomes and surrounding cytoplasm rather than a partial deterioration of the lipid membranes at the cellular and organelle levels.

### S-MGB uptake is not through an active transport mechanism

Active transport is an energy-dependent process, and previous studies have identified that sodium azide at concentrations of 0.2 mM to 10 mM can inhibit active transport *via* cytochrome *C* oxidase in mammalian cells.^33,34^ If the uptake of S-MGBs is reduced in the presence of sodium azide, then active transport could be identified as a potential mechanism responsible for S-MGB uptake. We therefore acquired Raman images of PNT2 and HeLa cells treated with each S-MGB (at a concentration of 10 μM or 20 μM) in the presence and absence of sodium azide (5 mM) (Fig. S8A, ESI†). Quantification of the alkyne signal intensities (Fig. S8B, ESI†) indicated that the uptake of both compounds in PNT2 cells and HeLa cells is not changed by sodium azide co-treatment.

**Fig. 3:**
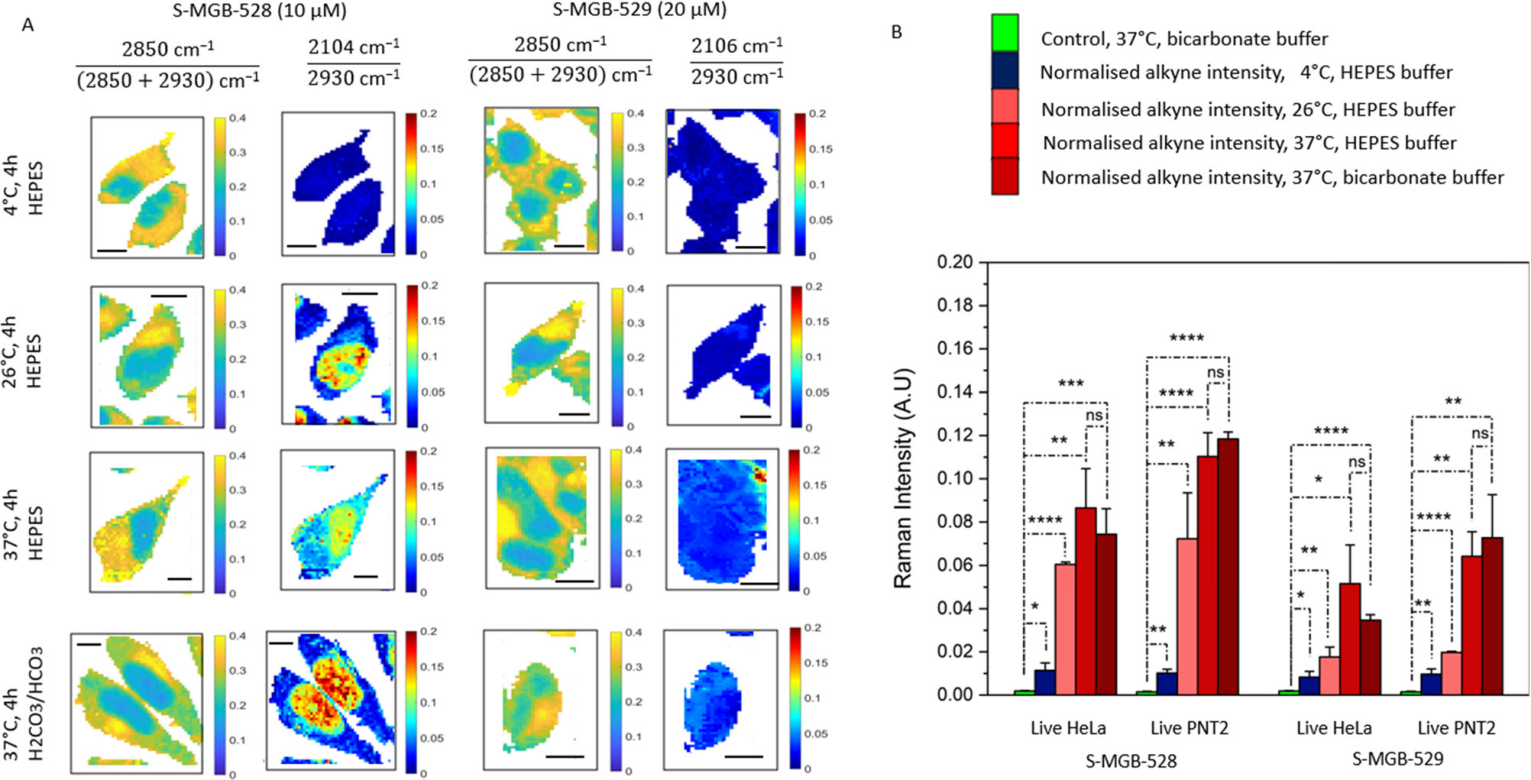
Investigating the temperature dependence of the S-MGBs uptake. (A) Representative Raman maps of live HeLa cells after the treatment with S-MGB-528 and S-MGB-529 (10/20 μM, 4 h, 4 °C/26 °C/37 °C, in presence of HEPES or bicarbonate buffer). The chemical contrast is shown by the ratio 2850 cm^−1^/(2850 + 2930) cm^−1^ indicative of CH_2_ and CH_3_ respectively. The drug localisation is shown through the normalised alkyne Raman intensity (*i.e.* 2104/2930 cm^−1^). (B) Normalised alkyne intensity of S-MGB-528 and S-MGB-529 in HeLa and PNT2 cells. The Raman data were collected using 532 nm, 0.5 s integration time, 10 mW laser power, 60× objective immersive lens, step size 1 μm in *x* and *y*, 1 accumulation. A minimum of three replicate images were acquired for each condition. Data represents mean ± SD (****p ≤ 0.0001; ***p ≤ 0.001; **p ≤ 0.01; *p ≤ 0.05; ns *p* > 0.05, Student's *t* test).

Active transport is also a temperature-dependent process, and is optimal at 37 °C.^35,36^ As such, we investigated the uptake of S-MGB-528 and S-MGB-529 in live PNT2 and HeLa cells at varying temperatures. We acquired Raman images of HeLa cells treated with either S-MGB (10 or 20 μM) at 4, 26, and 37 °C (Fig. 3A) and quantified the alkyne signal intensity in each case (Fig. 3B). A basal uptake for each S-MGB is observed at 4 °C by comparison with the DMSO controls (Fig. 3B). At 4 °C, the uptake of S-MGB-528 is reduced about 5- and 7-fold compared to 26 °C in HeLa and PNT2 cells, respectively (Fig. 3B). Alike, the uptake of S-MGB-529 is reduced about 2-fold in both HeLa and PNT2 cells. At 4 °C, the uptake of S-MGB-528 is reduced by about 7- and 11-fold compared to 37 °C in HeLa and PNT2 cells, respectively (Fig. 3B). Similarly, the uptake of S-MGB-529 is reduced by about 6- and 7-fold in HeLa and PNT2 cells, respectively (Fig. 3B). These data suggest that there is a temperature dependence in the uptake of both compounds in HeLa cells, and a similar effect was observed in PNT2 cells (Fig. S9, ESI†). The results also indicated that there is a basal level of drug uptake that is most likely due passive diffusion at 4 °C, given that the inhibition of active transport by NaN_3_ did not appear to impact S-MGB uptake. Together, these data indicate that the S-MGBs most likely enter the cells by passive diffusion rather than active transport (that would effectively be reduced to 0 at 4 °C) and that any differences in drug uptake are more likely due to changes in partition/distribution coefficients or different rates of diffusion which are both temperature-sensitive.^37^ We also investigated the effect of the buffer composition to see if osmolarity affected the intracellular uptake of S-MGBs. Changes in osmolarity would affect the intracellular uptake of the S-MGBs if a potential carrier exploits a secondary active transport mechanism (*i.e.* utilising Na^+^ gradient).^38^ The cells were treated with S-MGBs in either HEPES buffer (p*K*_a_ 7.3) or bicarbonate buffer (p*K*_a_ 6.3) at 37 °C (Fig. 3 and Fig. S9, ESI†). These experiments showed no statistical difference in the uptake concentration of either compound in both cell lines, indicating that the osmolarity of the buffer system has negligible effect on S-MGB uptake, confirming the absence of a secondary active transport mechanism being responsible for the overall uptake of the S-MGBs. Notably, cell-to-cell variation in S-MGB uptake most likely accounts for the minimal differences in nuclear accumulation in the cells incubated with HEPES or bicarbonate buffer, which in both cases, was shown to be not significant. Together, these experiments suggest that S-MGB uptake is not through an active transport mechanism.

### Facilitated diffusion is involved in S-MGB transport

**Fig. 4:**
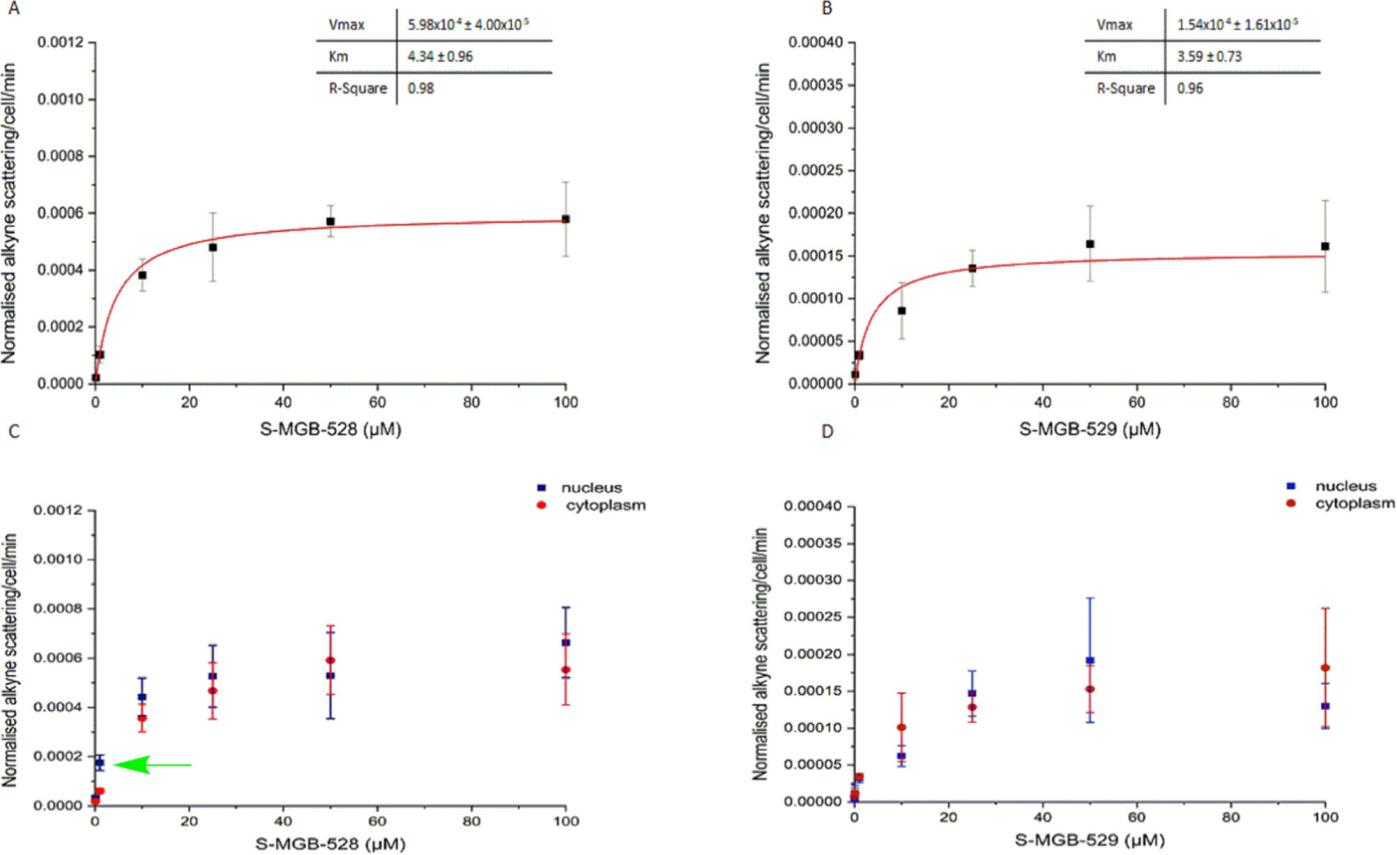
Investigating the kinetics of the uptake of the S-MGBs into live HeLa cells. Alkyne scattering of S-MGB-528 and S-MGB-529 in live HeLa cells for the whole cells (A and B) and cytoplasm and nuclear regions (C and D). HeLa cells were incubated with increasing concentration of either S-MGB-528 or S-MGB-529 (240 min). Following drug treatment and imaging, the alkyne Raman intensities for each concentration, and the whole cell, nucleus and cytoplasm, were measured and normalised to the symmetric stretching of the C–H at 2930 cm^−1^, then, the normalised Raman intensities were averaged and with the relative standard deviations divided by the time (240 min) to generate the kinetics of drug uptake associated with S-MGB-528 and S-MGB-529 in HeLa cells at the cellular and subcellular level. Lastly, the kinetics associated with the uptake of S-MGB-528 and S-MGB-529 and the relative standard deviation were plotted against the drug concentration fitting the Michaelis–Menten model whilst the kinetics of drug accumulation at the cytoplasmic and nuclear level were plotted against the drug concentration. Raman data were collected using 532 nm, 0.5 s integration time, 10 mW laser power, 60× immersive objective lens, step size 1 μm in *x* and *y*, 1 accumulation. A minimum of three replicate images were acquired for each condition. Data represents mean ± SD.

The previous experiments were based on the principle that both simple and facilitated diffusion are concentration dependent. However, unlike simple diffusion, it is possible that the process of facilitated diffusion may become saturated due to the involvement of an uptake carrier in the process. To test this hypothesis, we first investigated the potential saturation of the intracellular uptake of S-MGB-528 and S-MGB-529 in HeLa cells using a range of drug concentrations from 0 to 100 μM for 4 hours (Fig. 4). Both the compounds showed saturation in drug uptake indicating that involvement of a carrier in the internalisation of the S-MGBs in HeLa cells is probable (Fig. 4A and B). Similarly, in the case of PNT2 cells (Fig. S10, ESI†), both S-MGBs resulted in saturation in drug uptake indicating that facilitated diffusion is the probable mechanism responsible for drug uptake of S-MGBs in mammalian cells. Interestingly, S-MGB-528 alone showed a selective nuclear localisation at low concentration (1 μM, green arrow, Student's *t*-test *P* < 0.01), with negligible differences in uptake at higher concentrations (>5 μM) in HeLa cells (Fig. 4C and D). Meanwhile, in PNT2 cells, there was greater nuclear localisation at all concentrations tested. In addition, we used two structural analogues of the alkyne-labelled S-MGBs in which the alkyne is replaced by a methoxy group (Fig. S11, ESI†): a structural analogue of the S-MGB-528 (S-MGB-2) and a structural analogue of the S-MGB-529 (S-MGB-234). Co-treatment of the alkyne labelled S-MGBs and the structural analogues (used in excess) could potentially saturate any potential carrier protein that is required for facilitated diffusion. We acquired Raman images of HeLa cells treated with either S-MGB alone (10 μM or 20 μM) or HeLa cells treated with S-MGB (10 μM or 20 μM) and the structural analogues (at a fixed concentration of 100 μM) (Fig. S11B, ESI†). We quantified the alkyne signal intensity in each case (Fig. S11C, ESI†). A lower intracellular concentration of about 1.4-fold was measured for S-MGB-528 in HeLa cells (Fig. S11, ESI†) where drug co-treatment with the respective analogue had been used. In PNT2 cells, a lower intracellular concentration of about 2-fold was observed (Fig. S12, ESI†). Meanwhile, in the case of the amidine S-MGBs, the co-treatment with the structural analogue S-MGB-234 resulted in a lower uptake of S-MGB-529 in PNT2 cells, about 1.8-fold, but not in HeLa cells (Fig. S11C, ESI†). Overall, the co-treatment resulted in a more pronounced effect on the intracellular uptake of S-MGB-528 than S-MGB-529, and that this effect was less pronounced in HeLa cells. We speculate that changes in the intracellular uptake of S-MGB-529 in HeLa cells may not be possible to detect with these experimental conditions, and that the potential redundancy in the intracellular uptake of S-MGB-529 or S-MGB-234 in HeLa cells could have hidden the competitive diffusion of the molecules. These data are still consistent with uptake by facilitated diffusion, but perhaps with more than one carrier involved. Carriers which could be involved are Organic Cation Transport (OCT) carriers, although a further detailed investigation is beyond the scope of the current study.

Considering the results from our studies on uptake temperature, inhibition of active transport, and competition experiments, the uptake of both compounds in live PNT2 and HeLa cells likely involves a carrier that has similar affinity to S-MGB-528 and S-MGB-529 (due to the similar *K*_m_ values). However, the *V*_max_ value which is a rate of transport of the carrier substrate complex through the lipid membrane, is markedly different, with the likely greater lipophilicity associated with the S-MGB-528 carrier complex being responsible for a greater rate in intracellular uptake and drug cytotoxicity.^39^ Overall, facilitated diffusion is the more likely intracellular mode of uptake that S-MGBs exploit to enter mammalian cells.

### S-MGB-528 accumulates in the nucleus and then within lysosomes

**Fig. 5:**
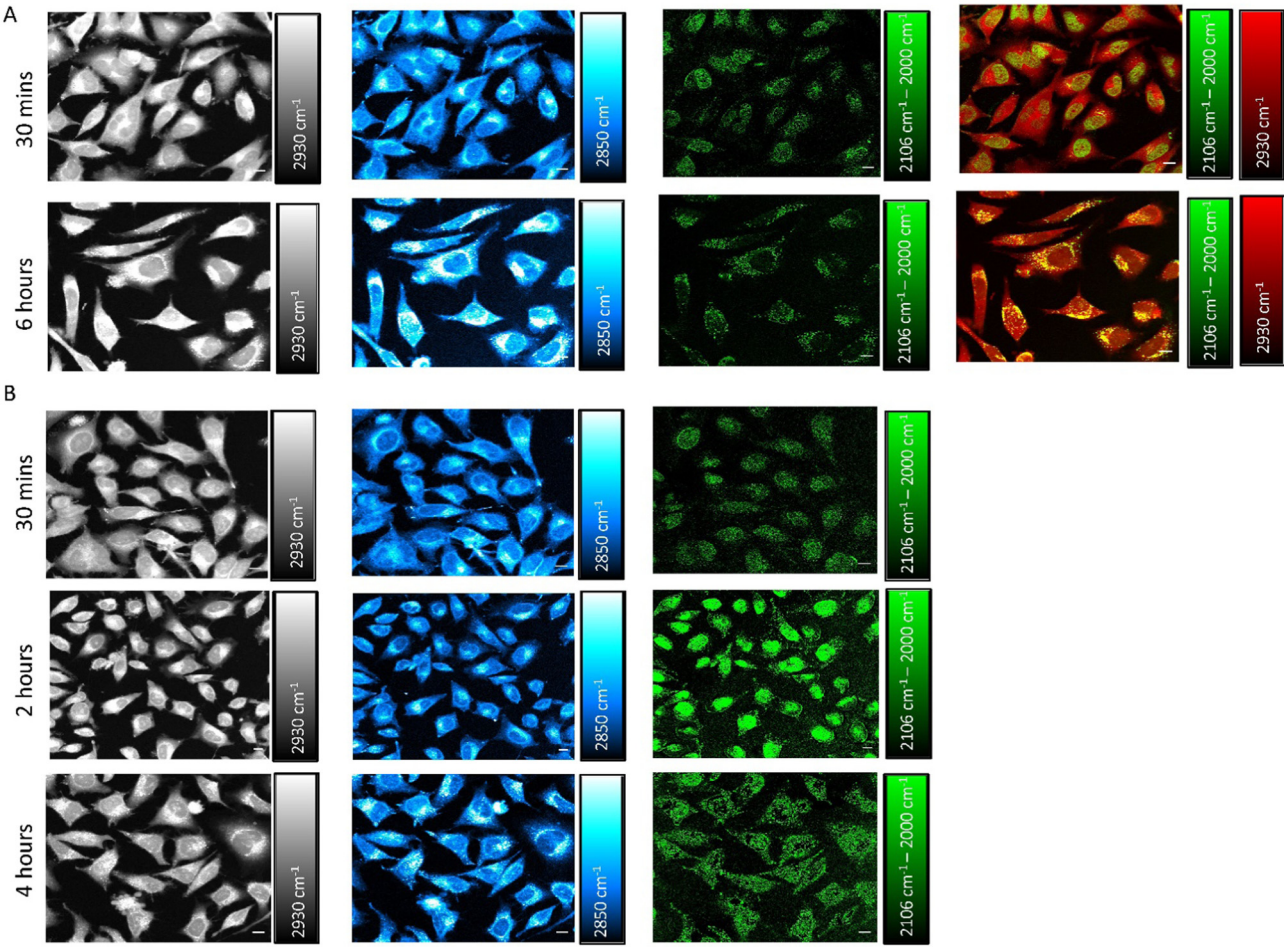
Investigating S-MGBs uptake using stimulated Raman scattering. (A) Time-dependent uptake of S-MGB-528. Representative SRS images of live HeLa cells after the treatment with S-MGB-528 (10 μM) for either 30 min or 6 h. The samples were imaged using SRS microscopy at the following: 2930 cm^−1^ (proteins), 2850 cm^−1^ (lipids), 2106 cm^−1^ (alkyne) and 2000 cm^−1^ (off-resonance). An image achieved by merging the channels of proteins and alkyne is also shown, highlighting the time-dependent subcellular localisation of the S-MGB-528. Lookup table: 0–3000 a.u. (Lipids and proteins), 0–1500 a.u. (on–off resonance) (B) The progressive lysosomal accumulation of S-MGB-528 in live HeLa cells using SRS microscopy. Representative SRS images of live HeLa cells after the treatment with S-MGB-528 (10 μM) for either 0.5, 2 and 4 h. The samples were imaged using SRS microscopy at the following: 2930 cm^−1^ (proteins), 2850 cm^−1^ (lipids) 2106 cm^−1^ (alkyne) and 2000 cm^−1^ (off-resonance). The time-lapse study highlights the progressive time-dependent accumulation of S-MGB 528 within the cytoplasm. Lookup table: 0–3000 a.u. (proteins and lipids), 0–1500 a.u. (on–off resonance). Scale bar size: 10 μm.

We next investigated the time-dependent uptake of S-MGB-528 into HeLa cells using SRS imaging, which enables fast-image acquisition across a larger field of view to study the effect of treatment time across a large number of cells with improved spatial resolution. SRS microscopy exploits two lasers, referred to as the pump and Stokes beams, that are overlapped in space and time to match the vibrational frequency of the chemical moiety of interest that is normally associated with the drug under investigation or with biomolecules, such as lipids and proteins. HeLa cells were treated with S-MGB-528 (10 μM) and SRS images were acquired in live cells after 30 min and 6 h treatment (Fig. 5A). SRS imaging was performed at 2930 cm^−1^ (CH_3_ symmetric stretch), 2850 cm^−1^ (CH_2_ symmetric stretch), 2106 cm^−1^ (=alkyne stretch) and 2000 cm^−1^ indicative of cellular proteins and lipids, and alkyne on- and off-resonance, respectively. SRS images of this type represent label-free markers of the cells and show after 6 h of treatment, an increase in the cellular lipids signal at 2850 cm^−1^. When imaging at 2106 cm^−1^ (C≡C, S-MGB-528) and 2000 cm^−1^ (off-resonance), a clear nuclear localisation of the on–off resonance SRS signal was detected at 30 min, whilst by 6 h, the predominant signal arises in the cytoplasm of the HeLa cells. A merged image of the on–off resonance SRS signal (alkyne, green) and the 2930 cm^−1^ (CH_3_, proteins, red) signal also highlighted the differences in subcellular localisation at the 30 min and 6 h timepoints. The distribution observed at 6 h suggests that the S-MGB-528 may selectively internalise at the organelle level; given that the morpholine group of S-MGB-528 has a p*K*_a_ ≈ 6, we hypothesised that lysosomes (pH ≈ 5) would be the most likely enrichment site. To investigate this, HeLa cells were treated with S-MGB-528 and LysoTracker Red that includes a multi-pyrrole conjugated fluorophore (BODIPY) and a weakly basic amine which leads to the lysosomal accumulation of the probe at the cellular level (Fig. S13, ESI†). Co-localization was confirmed by the merged image of the on–off resonance SRS signal (alkyne) signal and the LysoTracker Red signal after 6 h of treatment. A time-lapse study was further carried out to understand the initial lysosomal accumulation of S-MGB-528, suggesting that following 2 h of drug treatment, S-MGB-528 has already begun to accumulate in lysosomes (Fig. 5B). The observation that S-MGB-528 localises in the lysosome is consistent with the finding of other Raman-based studies of weakly-basic drugs including the tyrosine kinase inhibitors, ponatinib, nilotinib, imatinib and neratinib.^16–18^

We further investigated if the lysosomal distribution at the 6 h timepoint is due to nuclear efflux of S-MGB-528 or progressive intracellular drug accumulation from the culture medium, following the initial saturation of DNA (Fig. S14, ESI†). To do so, we treated live HeLa cells with S-MGB-528 for 30 min at a concentration of 10 μM, following which, the cells were washed and incubated in drug-free media. At the initial 30 min treatment, S-MGB-528 localised primarily in the nucleus and after the further incubation with drug-free media for 2, 4 and 6 h, only weak cytoplasmic alkyne signal was observed at the 6 h timepoint (Fig. S14, ESI†). These data suggested that saturation of the nucleus and then progressive intracellular drug accumulation as the most likely cause for the lysosomal trapping of S-MGB-528, rather than a redistribution from the nucleus to the lysosomes (Fig. 5). The weak cytoplasmic scattering observed at the 6 h timepoint is most likely a result of simple equilibrative diffusion.

Overall, we propose that there are two key factors involved in the subcellular localisation of these S-MGBs; the subcellular MGB concentration and the treatment time, and that these are related in the case of S-MGB-528. We hypothesise that the nucleus is the first and preferential location for these molecules. However, whilst this occurs within 30 min for S-MGB-528, a longer time period is required for S-MGB-529 to accumulate significantly at the nuclear level. The minor structural changes of the S-MGB tail motifs directly affect the biological properties of these compounds, altering their uptake concentrations and subcellular distribution.

## Conclusions

The purpose of this study was to investigate the uptake, distribution, and cytotoxicity of a pair of S-MGBs in mammalian cells using alkyne-tag Raman imaging. The specific S-MGBs under investigation were identical in structure except for the ‘tail group’, with S-MGB-528 bearing a morpholine and S-MGB-529 bearing an amidine. These different structural features were previously identified to modulate the cytotoxicity of S-MGBs, with compounds bearing an amidine tail group being notably less cytotoxic. The different physiochemical properties of the S-MGBs and the simultaneous presence of a carrier showing similar *K*_m_ but different *V*_max_ values for the S-MGBs may explain the observed intracellular uptake, distribution and cytotoxicity of the two compounds. With its higher affinity for the minor groove, S-MGB-529 may be expected to have a greater cytotoxicity than S-MGB-528; however, the opposite is true. This can now be explained as the Raman data presented here consistently identified a lower uptake of S-MGB-529 compared to S-MGB-528. This highlights the importance of the tail group in modulating cytotoxicity of S-MGBs, *via* relative intracellular accumulation, and the advantage of assessing S-MGBs in live cell studies, which take into account the cellular milieu.

Raman spectroscopy also provided evidence of a spectral red shift of the terminal alkyne group, which indicated the molecule was likely bound to the minor groove of DNA which is known to be locally hydrophobic. In addition, we have demonstrated the power of SRS microscopy for assessing the local distribution of S-MGB-528 with subcellular resolution and demonstrated that the S-MGB initially localised to the nucleus before accumulation of additional drug into lysosomes. Our results indicated that S-MGB-528 displays a greater uptake within both nuclear and lysosomal regions with a corresponding increased cytotoxicity when compared to S-MGB-529. Using metabolic inhibitors and structural analogues of the two minor groove binders, we successfully investigated the mechanism of S-MGB uptake into live cells and identified that facilitative diffusion was the most probable means by which the compounds are internalised. Importantly, it has been demonstrated that the significant changes in biological activity of S-MGBs upon small structural changes can be rationalised in terms of their differing underlying biological mechanisms. In this case, the importance of the amidine tail group in mitigating cytotoxicity is consistent with many ongoing studies showing that S-MGBs bearing an amidine tail group are well-tolerated *in vivo* and are suitable development candidates for new therapeutics.^20^ In addition, the use of structural analogues demonstrated the power of Raman spectroscopy for assessing binding efficiencies of the two drugs in a co-treatment experiment. To that end, our study reflects the potential of Raman microscopy for assessing the structure–activity–distribution features of novel molecules in the preclinical phase of drug discovery and highlights the clear advantage of investigating drug–cell interactions using an imaging-based approach.

## Experimental

### Cell culture: PNT2 cells

PNT2 cells were cultured in Roswell Park Memorial Institute medium (RPMI) supplemented with 1% penicillin/streptomycin, 1% amphotericin B and 10% heat-inactivated foetal bovine serum (FBS). Cells were incubated at 37 °C and 5% CO_2_ in a humified incubator. PNT2 cells were seeded (0.5 × 10^6^ per mL) on a coverslip (22 × 22 mm; #1.5H) in a 6-well plate for several measurements. After the overnight incubation, the media was removed and replaced with RPMI containing DMSO (control) or S-MGB at different concentrations (0.1–100 μM for either 4 or 24 h). For spontaneous Raman spectroscopy studies, PNT2 cells were seeded (*circa* 1 × 10^6^) in a total volume of 2 mL onto a coverslip (22 × 22 mm; #1.5H) in a 6-well plate. After the overnight incubation, the media was removed and replaced with RPMI containing DMSO (control) or Alk-MGB (0.1–100 μM). After a further 4 or 24 h of incubation, the medium was removed, the sample washed with PBS (3 × 2 mL) and imaged either as live or as a fixed sample in PBS buffer. The fixation step was performed by a 4% paraformaldehyde (PFA) solution in PBS (10 min, 37 °C, 5% CO_2_).

### HeLa cells

HeLa cells were cultured in Dulbecco's Modified Eagle's Medium (DMEM) supplemented with 1% penicillin/streptomycin, 1% amphotericin B and 10% heat-inactivated foetal bovine serum (FBS). Cells were incubated at 37 °C and 5% CO_2_ in a humified incubator. HeLa cells were seeded (0.5 × 10^6^ per mL) on a coverslip (22 × 22 mm; #1.5H) in a 6-well plate for several measurements. After the overnight incubation, the media was removed and replaced with DMEM containing DMSO (control) or S-MGB at different concentrations (0.1–100 μM for either 4 or 24 h). For spontaneous Raman spectroscopy studies, HeLa cells were seeded (*circa* 1 × 10^6^) in a total volume of 2 mL on a coverslip (22 × 22 mm; #1.5H) in a 6-well plate. After the overnight incubation, the media was removed and replaced with DMEM containing DMSO (control) or S-MGB (0.1–100 μM). After a further 4 h or 24 h of incubation, the medium was removed, the sample washed with PBS (3 × 2 mL) and imaged either live or fixed in PBS buffer. The fixation step was performed by a 4% paraformaldehyde (PFA) solution in PBS (10 min, 37 °C, 5% CO_2_). For the stimulated Raman scattering studies, HeLa cells were seeded (*circa* 1 × 10^6^) in a total volume of 2 mL on a coverslip (22 × 22 mm; #1.5H) in a 6-well plate. After the overnight incubation, the media was removed and replaced with DMEM containing DMSO (control) or S-MGB (10 μM). After incubation for the indicated timepoints, the medium was removed, the sample washed with PBS (3 × 2 mL), mounted onto a glass microscope slide using a PBS boundary as reported previously, and imaged live.^16^

### Thermal melt experimental

Salmon genomic DNA (gDNA; D1626, Sigma-Aldrich) at 1 mg mL^−1^ in 1 mM phosphate buffer (pH 7.4) containing 0.27 mM KCl and 13.7 mM NaCl (P4417, Sigma-Aldrich) was annealed at 90 °C for 10 min and left to cool to room temperature. S-MGBs at 10 mM in DMSO were diluted with the same phosphate buffer to yield a single sample with 10 μM S-MGB and 0.02 mg mL^−1^ gDNA in 1 mM phosphate buffer containing 0.27 mM KCl and 13.7 mM NaCl. Control samples containing only S-MGB or gDNA were prepared, respectively. Samples were melted at a rate of 0.5 °C min^−1^ from 45 °C to 90 °C with spectra recorded at 260 nm on a UV-1900 UV-vis spectrophotometer fitted with a Peltier temperature controller (Shimadzhu) using LabSolutions (Tm Analysis) software. The melting temperatures (*T*_m_s) of the S-MGB:DNA complexes were determined by fitting a sigmoidal function using a Boltzmann distribution in OriginPro. Two independent experiments were carried out with values quoted with an error no worse than ±0.5 °C.

### Raman measurements

All Raman spectra and images were acquired on a Renishaw inVia Raman microscope controlled by WiRE 4.4 software. The system was equipped with a 532 nm Nd:YAG laser and providing a maximum output of 50 mW and using a 1800 lines per mm grating, and a 785 nm diode laser providing a maximum output of 300 mW and using a 1200 lines per mm grating. Raman analysis was performed using a 5×, 20×, 50× or 100× objective lens (Leica Microsystems) or a 60× water immersion lens (Nikon). Prior to spectral acquisitions, the instrument was calibrated using the internal silicon standard at 520.5 cm^−1^. Raman spectra of the solid S-MGBs were acquired from a small amount of the solid which was transferred to a calcium fluoride disc (Crystran, UK) using 785 nm laser excitation with a 5× lens (*ca.* 7.8 mW) for 10 s. Raman spectra of S-MGBs in solution were acquired using 532 nm laser excitation using a 60× objective lens (*ca.* 10 mW) with 1 accumulation (determining intracellular concentration), 10 accumulations (environmental sensing experiment in DMSO: aqueous solutions) for 0.5 s. Raman mapping experiments were acquired using 532 nm laser excitation with a 60× objective lens (*ca.* 10 mW) for 0.5 s.

### SRS imaging

A schematic diagram of the SRS microscope used in this study is presented in Fig. S15 (ESI†). An integrated laser system (picoEmerald S, Applied Physics & Electronics, Inc.) was used to produce two synchronised laser beams at 80 MHz repetition rate. A fundamental Stokes beam (1031.2 nm, 2 ps pulse width) was intensity modulated by an electro-optic-modulator with >90% modulation depth, and a tunable pump beam (700–990 nm, 2 ps pulse width, <1 nm (∼10 cm^−1^) spectral bandwidth) was produced by a built-in optical parametric oscillator. The pump and Stokes beams were spatially and temporally overlapped using two dichroic mirrors and a delay stage inside the laser system and coupled into an inverted laser-scanning microscope (Leica TCS SP8, Leica Microsystems) with optimised near-IR throughput. SRS images were acquired using a 40× objective (HC PL IRAPO 40, N.A. 1.10 water immersion lens) with a 9.75–48 μs pixel dwell time over a 512 × 512 or a 1024 × 1024 frame. The Stokes beam was modulated with a 20 MHz EoM. Forward scattered light was collected by a S1 N.A. 1.4 condenser lens (Leica Microsystems). To acquire SRS images, the pump beam intensity is recorded in the forward direction using a silicon photodiode and demodulated using a lock-in amplifier (Zurich Instruments). Images were acquired at 12-bit image depth. The laser powers measured after the objective lens were in the range 10–30 mW for the pump beam only, 10–50 mW for the Stokes beam only and 20–70 mW (pump and Stokes beams). Samples were imaged at different Raman shifts, such as 2930 cm^−1^, 2850 cm^−1^, 2106 cm^−1^ or 2000 cm^−1^ (off-resonance).

### Temperature dependent uptake of S-MGBs

PNT2 and HeLa cells were treated with S-MGB-528 (10 μM, 4 h) and S-MGB-529 (20 μM, 4 h) in either RPMI or DMEM containing HEPES (15 mM) at different temperatures (4 °C, 26 °C and 37 °C). The analysis also included a negative control (DMSO) and a sample incubated with DMEM or RPMI containing S-MGB-528 or S-MGB-529 in the absence of HEPES, to investigate any change in intracellular uptake in dependence of the osmolarity of the medium. Following the incubation, the cells were washed with PBS (3 × 2 mL) and imaged in PBS (4 mL).

### Saturation of the intracellular uptake

PNT2 and HeLa cells were treated with RPMI or DMEM containing S-MGB-528 or S-MGB-529 at increasing concentrations (0.1–100 μM) for 4 h. Following the incubation, the cells were washed with PBS (3 × 2 mL) and imaged in PBS (4 mL).

### The intracellular uptake in presence of a competitor

To investigate the presence of facilitated diffusion, PNT2 and HeLa cells were treated with S-MGB-528 (10 μM, 4 h) and S-MGB-529 (20 μM, 4 h) in either RPMI or DMEM in the presence of structural analogue, S-MGB-2 (100 μM) and MGB-234 (100 μM), respectively. Following the incubation, the cells were washed with PBS (3 × 2 mL) and imaged in PBS (4 mL).

### Washout experiment with S-MGB-528

To demonstrate the persistent nuclear location of the S-MGB-528 with time, HeLa cells were treated with S-MGB-528 (10 μM, 30 min) and then further incubated with fresh DMEM in the absence of S-MGB-528 for up to 6 h. The cells were washed with PBS (3 × 2 mL) and imaged using SRS microscopy.

### Multimodal imaging of S-MGB-528 and LysoTracker Red

To demonstrate the lysosomal location, following the washing with PBS (2 mL × 3), all the samples were incubated with DMEM containing with LysoTracker Red DND 99 (50 nM, 30 min) and imaged live without any additional washing steps as per manufacturer's guideline. Fluorescence images of LysoTracker Red signal were acquired using *λ*_ex_ = 561 nm, *λ*_em_ = 570–650 nm.

### Data processing and analysis

The Raman spectral data were pre-processed using WiRE 4.4 software. For the solid spectra of S-MGB-528 and S-MGB-529, this process involved the removal of the cosmic rays followed by the smoothing and the baseline subtraction using polynomial fitting. Raman spectra collected from PNT2 and HeLa cells were pre-processed by Wire 4.4. This process involved the truncation of the Raman spectrum (1850–3100 cm^−1^), the removal of the cosmic rays using a nearest neighbour algorithm, noise filtering, smoothing and baseline subtraction using a polynomial fitting.

The pre-processed data was imported into Matlab2020 and ratiometric analysis was performed using a modified script that has been reported previously.^29^ This method was used to create false colour ratiometric images from the Raman mapping data. A false colour map of the ratio intensity: 2106/2930 cm^−1^ was created to map the presence of the alkyne in the cell. Nuclear and cytoplasmic regions were discriminated by a ratiometric approach; false colour maps created by the ratio of the peaks: 2850 cm^−1^/(2850 cm^−1^ + 2930 cm^−1^) were created. The script generated the average Raman spectra from all pixels and the average Raman spectra from the segmented regions-of-interest (the nucleus and cytoplasm based on the ratios previously described).

The kinetics associated with either S-MGB uptake for the whole cell were achieved by fitting the means and standard deviations of the normalised alkyne Raman intensity for a Michaelis–Menten model (Origin 2022). Alternatively, *V*_max_ and *K*_m_ for the whole cells were calculated from the software.

### Ratiometric analysis of Raman images

Ratiometric analysis has previously been used to identify the cellular nucleus and cytoplasm based on the ratio of the Raman bands at 2850 cm^−1^/(2850 + 2930 cm^−1^). The ratiometric analysis was performed on the Raman images using a custom Matlab script reported previously (ref. 29).

### Image analysis

SRS images were processed using ImageJ software. Briefly, consistent brightness/contrast settings were applied, scale bars and false colours were applied using ImageJ. The SRS images acquired at 2106 cm^−1^ (alkyne, S-MGB) were background subtracted with an off-resonance image (2000 cm^−1^) using the Image Calculator function.

### Viability study

To investigate the cytotoxicity of S-MGB-528 and S-MGB-529 in mammalian cells, PNT2 and HeLa cells (0.5 × 10^6^ mL) were seeded and incubated in fresh RPMI or DMEM for 24 h (37 °C, 5% CO_2_). The medium was removed, and the cells treated with RPMI or DMEM containing S-MGB-528 or S-MGB-529 at increasing concentrations (0.1–50 μM) for 24 h. Following the incubation, the cells were washed with PBS (3 × 2 mL), detached with 0.05% trypsin:EDTA (1 mL, 5 min at 37 °C) and collected by adding a further 1 mL of culture medium. The viable cells were counted using Trypan Blue stain (0.4% in PBS). The viable cells were expressed as a percentage of the DMSO control (100%).

## Author contributions

C. T. performed the Raman imaging analysis. C. T. and W. J. T. performed all data analysis and wrote the manuscript. L. McG. and L. M. B. synthesised the S-MGBs. L. McG. performed the thermal shift assays. L. McG. and I. T.-H. performed native mass spectrometry analysis. C. T. was responsible for cell culture. C. W. was the cell-lab manager. S. L., C. J. S., K. F., F. J. S., R. B. and D. G. supervised the project, drafted the manuscript and are responsible for funding.

## Conflicts of interest

F. J. S. and C. J. S. are inventors on patents that pertain to S-MGBs, filed by the University of Strathclyde, and may benefit financially from commercial efforts relating to these patents.

## Supplementary Material

CB-003-D2CB00159D-s001

## References

[cit1] Suckling C. J. (2004). Minor Groove Binders 1998–2004. Expert Opin. Ther. Pat..

[cit2] Nelson S. M., Ferguson L. R., Denny W. A. (2007). Non-Covalent Ligand/DNA Interactions: Minor Groove Binding Agents. Mutat. Res., Fundam. Mol. Mech. Mutagen..

[cit3] Wilson W. D., Nguyen B., Tanious F. A., Mathis A., Hall J. E., Stephens C. E., Boykin D. W. (2005). Dications That Target the DNA Minor Groove: Compound Design and Preparation, DNA Interactions, Cellular Distribution and Biological Activity. Curr. Med. Chem. – Anti-Cancer Agents.

[cit4] Khalaf A. I., Al-Kadhimi A. A. H., Ali J. H. (2016). DNA Minor Groove Binders-Inspired by Nature. Acta Chim. Slov..

[cit5] Barrett M. P., Gemmell C. G., Suckling C. J. (2013). Minor Groove Binders as Anti-Infective Agents. Pharmacol. Ther..

[cit6] Ungogo M. A., Campagnaro G. D., Alghamdi A. H., Natto M. J., de Koning H. P. (2022). Differences in Transporters Rather than Drug Targets Are the Principal Determinants of the Different Innate Sensitivities of Trypanosoma Congolense and Trypanozoon Subgenus Trypanosomes to Diamidines and Melaminophenyl Arsenicals. Int. J. Mol. Sci..

[cit7] Carter N. S., Berger B. J., Fairlamb A. H. (1995). Uptake of Diamidine Drugs by the P2 Nucleoside Transporter in Melarsen-Sensitive and -Resistant Trypanosoma Brucei Brucei. J. Biol. Chem..

[cit8] Benton C. B., Diaz-Pavon J. R., Maiti A., Daver N. G., Ravandi F., Jain N., Alvarado Y., Jabbour E., Pierce S., Kwari M., Santos M. A., Martinez S., Siguero M., Tefferi A., Cortes J. E., Kantarjian H. M., Pardanani A. D., Garcia-Manero G. (2017). Phase I Study of Lurbinectedin (PM11083) in Patients with Advanced AML and MDS. J. Clin. Oncol..

[cit9] FDA. FDA grants accelerated approval to lurbinectedin for metastatic small cell lung cancer https://www.fda.gov/drugs/drug-approvals-and-databases/fda-grants-accelerated-approval-lurbinectedin-metastatic-small-cell-lung-cancer

[cit10] Conway J. R. W., Carragher N. O., Timpson P. (2014). Developments in Preclinical Cancer Imaging: Innovating the Discovery of Therapeutics. Nat. Rev. Cancer.

[cit11] Buchberger A. R., DeLaney K., Johnson J., Li L. (2018). Mass Spectrometry Imaging: A Review of Emerging Advancements and Future Insights. Anal. Chem..

[cit12] Lang W., Yuan C., Zhu L., Du S., Qian L., Ge J., Yao S. Q. (2020). Recent Advances in Construction of Small Molecule-Based Fluorophore-Drug Conjugates. J. Pharm. Anal..

[cit13] Belitsky J. M., Leslie S. J., Arora P. S., Beerman T. A., Dervan P. B. (2002). Cellular Uptake of N -Methylpyrrole/N-Methylimidazole Polyamide-Dye Conjugates. Bioorg. Med. Chem..

[cit14] Narayanaswamy N., Das S., Samanta P. K., Banu K., Sharma G. P., Mondal N., Dhar S. K., Pati S. K., Govindaraju T. (2015). Sequence-Specific Recognition of DNA Minor Groove by an NIR-Fluorescence Switch-on Probe and Its Potential Applications. Nucleic Acids Res..

[cit15] Tipping W. J., Lee M., Serrels A., Brunton V. G., Hulme A. N. (2016). Stimulated Raman Scattering Microscopy: An Emerging Tool for Drug Discovery. Chem. Soc. Rev..

[cit16] Fu D., Zhou J., Zhu W. S., Manley P. W., Wang Y. K., Hood T., Wylie A., Xie X. S. (2014). Imaging the Intracellular Distribution of Tyrosine Kinase Inhibitors in Living Cells with Quantitative Hyperspectral Stimulated Raman Scattering. Nat. Chem..

[cit17] Sepp K., Lee M., Bluntzer M. T. J., Helgason G. V., Hulme A. N., Brunton V. G. (2020). Utilizing Stimulated Raman Scattering Microscopy to Study Intracellular Distribution of Label-Free Ponatinib in Live Cells. J. Med. Chem..

[cit18] Aljakouch K., Lechtonen T., Yosef H. K., Hammoud M. K., Alsaidi W., Kötting C., Mügge C., Kourist R., El-Mashtoly S. F., Gerwert K. (2018). Raman Microspectroscopic Evidence for the Metabolism of a Tyrosine Kinase Inhibitor, Neratinib, in Cancer Cells. Angew. Chem., Int. Ed..

[cit19] Scott F. J., Khalaf A. I., Giordani F., Wong P. E., Duffy S., Barrett M., Avery V. M., Suckling C. J. (2016). An Evaluation of Minor Groove Binders as Anti-Trypanosoma Brucei Brucei Therapeutics. Eur. J. Med. Chem..

[cit20] Giordani F., Khalaf A. I., Gillingwater K., Munday J. C., De Koning H. P., Suckling C. J., Barrett M. P., Scott F. J. (2019). Novel Minor Groove Binders Cure Animal African Trypanosomiasis in an in Vivo Mouse Model. J. Med. Chem..

[cit21] Scott F. J., Khalaf A. I., Duffy S., Avery V. M., Suckling C. J. (2016). Selective Anti-Malarial Minor Groove Binders. Bioorg. Med. Chem. Lett..

[cit22] Scott F. J., Nichol R. J. O., Khalaf A. I., Giordani F., Gillingwater K., Ramu S., Elliott A., Zuegg J., Duffy P., Rosslee M. J., Hlaka L., Kumar S., Ozturk M., Brombacher F., Barrett M., Guler R., Suckling C. J. (2017). An Evaluation of Minor Groove Binders as Anti-Fungal and Anti-Mycobacterial Therapeutics. Eur. J. Med. Chem..

[cit23] Suckling C. , Chemistry – the Queen of Sciences Heterocyclic chemistry in drug discovery at the university of Strathclyde https://www.mgb-biopharma.com/wp-content/uploads/Prof-Colin-Suckling-ebook-v3.pdf

[cit24] Khalaf A. I., Bourdin C., Breen D., Donoghue G., Scott F. J., Suckling C. J., MacMillan D., Clements C., Fox K., Sekibo D. A. T. (2012). Design, Synthesis and Antibacterial Activity of Minor Groove Binders: The Role of Non-Cationic Tail Groups. Eur. J. Med. Chem..

[cit25] Breen D., Kennedy A. R., Suckling C. J. (2009). A Divergent Synthesis of Minor Groove Binders with Tail Group Variation. Org. Biomol. Chem..

[cit26] Wojcik K., Dobrucki J. W. (2008). Interaction of a DNA Intercalator DRAQ5, and a Minor Groove Binder SYTO17, with Chromatin in Live Cells - Influence on Chromatin Organization and Histone - DNA Interactions. Cytom. Part A.

[cit27] Peng X., Wu T., Fan J., Wang J., Zhang S., Song F., Sun S. (2011). An Effective Minor Groove Binder as a Red Fluorescent Marker for Live-Cell DNA Imaging and Quantification. Angew. Chem..

[cit28] Scott F. J., Puig-Sellart M., Khalaf A. I., Henderson C. J., Westrop G., Watson D. G., Carter K., Grant M. H., Suckling C. J. (2016). An Evaluation of Minor Groove Binders as Anti-Lung Cancer Therapeutics. Bioorg. Med. Chem. Lett..

[cit29] Jamieson L. E., Wetherill C., Faulds K., Graham D. (2018). Ratiometric Raman Imaging Reveals the New Anti-Cancer Potential of Lipid Targeting Drugs. Chem. Sci..

[cit30] Suckling C. J. (2015). The Antibacterial Drug MGB-BP3: From Discovery to Clinical Trial. Chem. Biol. Interface.

[cit31] Nichol R. J. O., Khalaf A. I., Sooda K., Hussain O., Griffiths H. B. S., Phillips R., Javid F. A., Suckling C. J., Allison S. J., Scott F. J. (2019). Selective: In Vitro Anti-Cancer Activity of Non-Alkylating Minor Groove Binders. MedChemComm.

[cit32] Neidle S. (2001). DNA Minor-Groove Recognition by Small Molecules. Nat. Prod. Rep..

[cit33] Monti D. M., Guarnieri D., Napolitano G., Piccoli R., Netti P., Fusco S., Arciello A. (2015). Biocompatibility, Uptake and Endocytosis Pathways of Polystyrene Nanoparticles in Primary Human Renal Epithelial Cells. J. Biotechnol..

[cit34] Li Y., Monteiro-Riviere N. A. (2016). Mechanisms of Cell Uptake, Inflammatory Potential and Protein Corona Effects with Gold Nanoparticles. Nanomedicine.

[cit35] Fiorentino I., Gualtieri R., Barbato V., Mollo V., Braun S., Angrisani A., Turano M., Furia M., Netti P. A., Guarnieri D., Fusco S., Talevi R. (2015). Energy Independent Uptake and Release of Polystyrene Nanoparticles in Primary Mammalian Cell Cultures. Exp. Cell Res..

[cit36] Seidel J., Miao Y., Porterfield W., Cai W., Zhu X., Kim S. J., Hu F., Bhattarai-Kline S., Min W., Zhang W. (2019). Structure-Activity-Distribution Relationship Study of Anti-Cancer Antimycin-Type Depsipeptides. Chem. Commun..

[cit37] Sugano K., Kansy M., Artursson P., Avdeef A., Bendels S., Di L., Ecker G. F., Faller B., Fischer H., Gerebtzoff G., Lennernaes H., Senner F. (2010). Coexistence of Passive and Carrier-Mediated Processes in Drug Transport. Nat. Rev. Drug Discovery.

[cit38] Andreev E., Brosseau N., Carmona E., Mes-Masson A. M., Ramotar D. (2016). The Human Organic Cation Transporter OCT1 Mediates High Affinity Uptake of the Anticancer Drug Daunorubicin. Sci. Rep..

[cit39] Tonkin K. C., Brownlee R. T. C., Zunino F., Phillips D. R. (1990). Fluorinated Anthracyclines: Synthesis and Biological Activity. Invest. New Drugs.

