# Supplementary material for: Ratiometric imaging of minor groove binders in mammalian cells using Raman microscopy
Source: RSC Chem Biol. 2022 Sep 26;3(12):1403–15. doi: 10.1039/d2cb00159d (PMC9709774; doi:10.1039/d2cb00159d)
Supplement: CB-003-D2CB00159D-s001 [file CB-003-D2CB00159D-s001.pdf]

Electronic Supplementary Material (ESI) for RSC Chemical Biology.
This journal is © The Royal Society of Chemistry 2022

Ratiometric imaging of minor groove binders in mammalian cells using Raman microscopy

Electronic Supplementary Information

Christian Tentellino,[a] William J. Tipping,[a] Leah M. C. McGee,[b] Laura M. Bain,[b] Corinna Wetherill,[a] Stacey Laing,[a] Izaak Tyson Hirst,[b] Colin J. Suckling,[b] Rebecca Beveridge,[b] Fraser J. Scott,[b] Karen Faulds*[a] and Duncan Graham*[a]*

[a] *Centre for Molecular Nanometrology, WestCHEM, Department of Pure and Applied Chemistry, Technology and Innovation Centre, University of Strathclyde, 99 George Street, Glasgow G1 1RD, UK.*

[b] *Department of Pure and Applied Chemistry, University of Strathclyde, 295 Cathedral Street, Glasgow G1 1XL, UK.*

Contents

Materials and Methods……………………………………………………………………………2

Figure S1-S15…………………………………………………………………………………11

Materials and Methods.

Thermal Melt Experimental

Salmon genomic DNA (gDNA; D1626, Sigma–Aldrich) at 1 mg/mL in 1 mM phosphate buffer (pH 7.4) containing 0.27 mM KCl and 13.7 mM NaCl (P4417, Sigma–Aldrich) was annealed at 90°C for 10 min and left to cool to room temperature. S-MGBs at 10 mM in DMSO were diluted with the same phosphate buffer to yield a single sample with 10 μM S-MGB and 0.02 mg/mL gDNA in 1 mM phosphate buffer containing 0.27 mM KCl and 13.7 mM NaCl. Control samples containing only S-MGB or gDNA were prepared, respectively. Samples were melted at a rate of 0.5°C/min from 45°C to 90°C with spectra recorded at 260 nm on a UV-1900 UV-vis spectrophotometer fitted with a Peltier temperature controller (Shimadzhu) using LabSolutions (Tm Analysis) software. The melting temperatures (Tms) of the S-MGB:DNA complexes were determined by fitting a sigmoidal function using a Boltzmann distribution in OriginPro. Two independent experiments were carried out with values quoted with an error no worse than +1°C.

Mass spectroscopy experiment

DNA Sample Preparation

DNA oligonucleotide sequence 5'-CGCATATATGCG-3' was purchased in lyophilized form from Alpha DNA, Canada) and used without further purification, purity assessed by NMR. 100 μM stock solutions of DNA were prepared with 150 mM ammonium acetate buffer solution (Fisher Scientific, Loughborough, Leicestershire, UK) and 2 mM potassium chloride solution (Fisher Scientific, Loughborough, Leicestershire, UK). This solution was annealed at 90 degrees for 10 minutes and allowed to cool to room temperature. 10 mM S-MGB stock in 100% DMSO (Sigma-Aldrich, St. Louis, MO, USA) were diluted to 1 mM S-MGB solution with 150 mM ammonium acetate. Final samples were prepared from this solution to yield final concentrations of 9 *μ*M DNA, 100 *μ*M KCl, and 100 *μ*M s-MGB, 1% DMSO. DNA solutions containing no S-MGB included 1 % DMSO and were used as controls.

Mass Spectrometry Measurements

Native mass spectrometry experiments were carried out on a Synapt G2Si instrument (Waters, Manchester, UK) with a nanoelectrospray ionization source (nESI). Mass calibration was performed by a separate infusion of NaI cluster ions. Solutions were ionized from a thin-walled borosilicate glass capillary (i.d. 0.78 mm, o.d. 1.0 mm, (Sutter Instrument Co., Novato, CA, USA) pulled in-house to nESI tip with a Flaming/Brown micropipette puller (Sutter Instrument Co., Novato, CA, USA). A negative potential in range of 1.0 kV – 1.2 kV was applied to the solution via a thin platinum wire (diameter 0.125 mm, Goodfellow, Huntingdon, UK). The following instrument parameters were used for the DNA: S-MGB-528 complex: capillary voltage 1.1 kV, sample cone voltage 90 V, source offset 110 V, source temperature 40 °C, trap collision energy 3.0 (V), trap gas 5 mL/min. For DNA: S-MGB-529 complex: capillary voltage 1.2 kV, sample cone voltage 90 V, source offset 110 V, source temperature 40 °C, trap collision energy 5.0 (V), trap gas 5 mL/min was used. For DNA with no MGB present, a capillary voltage 1.0 kV was applied to the sample. Sample cone voltage 80 V, source offset 95 V, source temperature 40 °C, trap collision energy 3.0 (V) and trap gas 4.0 mL/min was used. Data were processed using Masslynx V4.2 and OriginPro 2021, and figures were produced using chemdraw.

Raman spectroscopy

All Raman spectra were acquired on a Renishaw inVia Raman microscope equipped with a 532 nm Nd:YAG laser providing a maximum output of 50 mW and using a 1800 lines per mm grating, and a 785 nm diode laser providing a maximum output of 300 mW using a 1200 lines per mm grating. Prior to spectral acquisitions, the instrument was calibrated using the internal silicon standard at 520.5 cm^{-1}.

Investigating the alkyne Raman shift of S-MGBs in aqueous: organic mixtures

To investigate the responsiveness of S-MGB-528 and S-MGB-529 to the chemical composition, the alkyne Raman scattering of the compounds was investigated in aqueous:DMSO mixtures. S-MGBs were prepared at a concentration of 20 mM in water:DMSO (100:0 to 0:100 v/v). The solutions were transferred to a perfusion chamber system (Grace BioLabs) and Raman spectra were acquired using 532 nm excitation with a 60× lens (ca. 10 mW) for 0.5 s and 20 accumulations. Three replicate spectra were acquired per concentration.

Determining intracellular uptake concentration of S-MGBs

The alkyne Raman scattering of S-MGB-528 and S-MGB-529 in PBS solution, in the range of concentration between 0 and 52 mM, was measured. The measurements were performed using a perfusion chamber system (Grace BioLabs) under equivalent imaging conditions used for live cells. The measured linear relationship was then used to generate the equation used to estimate the *in cellulo* concentration of the compounds.

Intracellular uptake of S-MGBs using cellular permeabilisation

To investigate the role of the lipid membrane on the intracellular drug uptake, PNT2 and HeLa cells were seeded according to the Materials and Methods section, before washing with PBS (3×2 mL), fixation with 4% PFA in PBS (2 mL, 10 mins, 37°C) before further washing with PBS (2 mL x 3). The cells were either incubated with PBS in the presence or absence of TRITON-X-100 (0.1% v/v) for 10 mins before wasing with PBS (2 mL x 3). The cells were then incubated with DMEM or RPMI containing S-MGB-528 or S-MGB-529 (10 µM, 10 mins, room temperature) before washing with PBS (2 mL x 3) and imaging in PBS (4 mL).

Intracellular uptake in the presence of the metabolic inhibitor, NaN_3

To investigate the presence of active transport, PNT2 and HeLa cells were treated with S-MGB-528 (10 µM, 4 h) and S-MGB-529 (20 µM, 4 h) in either RPMI or DMEM in the presence or absence of NaN_3 (5 mM). Following the incubation, the cells were washed with PBS (3×2 mL) and imaged in PBS (4 mL).

The intracellular uptake in presence of a competitor

To investigate the presence of facilitated diffusion, PNT2 and HeLa cells were treated with S- MGB-528 (10 µM, 4 h) and S-MGB-529 (20 µM, 4 h) in either RPMI or DMEM in presence of structural analogue, S-MGB-2 (100 µM) and S-MGB-234 (100 µM), respectively. Following the incubation, the cells were washed with PBS (3×2 mL) and imaged in PBS (4 mL).

General methods for the synthesis of the S-MGBs

Nuclear Magnetic Resonance (NMR) Experiments

All ^{1}H NMR and ^{13}C NMR spectral data for synthesised compounds were recorded using a Bruker DRX 500 spectrometer at 500 and 126 Hz respectively, with console advance III HD and 4.0.7 topspin software, using the deuterated solvent specified. Chemical shift values (δ) are expressed in parts per million (ppm). The following abbreviations are used for the multiplicity of the ^{1}H NMR signals: s (singlet), d (doublet), dd (doublet of doublets), dt (doublet of triplets), ddd doublet of doublets of doublets), m (multiplet), t (triplet), td (triplet of doublets), and q (quartet). Coupling constants are listed as J values, measured in Hz. Solvent references were DMSO-d_6 referenced at 2.50 (^{1}H) and 39.52 ppm (^{13}C).

Infrared (IR) spectroscopy

IR spectra were run on a Shimadzu Corp. IRAffinity-1S Fourier Transform Infrared spectrophotometer fitted with a single reflection ATR accessory.

Freeze Dryer

Samples contained within an appropriately sized round bottomed flask were frozen by submerging in liquid nitrogen before being lyophilized using an LTE Mini Lyotrap Freeze Dryer.

HPLC Purification

When appropriate, compounds were purified via HPLC chromatography using a Gilson PL C2250, fitted with a custom 5 µm particle size Nucleodur C18 (50x40 – 75 g) Gravity Preparative Column. Flow rate 25 mL/min. Solvent A (Water w/0.1% TFA), Solvent B (Acetonitrile w/0.1% TFA).

Table S1: HPLC purification method run 1.

Time	Flow Rate	% Solvent A	% Solvent B
**00 s**	25	75	25
**11.50**	25	70	30
**30.10**	25	40	60
**40.00**	25	80	20

Table S2: HPLC purification method run 2.

Time	Flow Rate	% Solvent A	% Solvent B
**00 s**	25	95	05
**5.00**	25	90	10
**25.00**	25	50	50
**30.00**	25	50	50
**35.00**	25	30	70
**40.00**	25	95	05

Low-Resolution Mass Spectra (LR-MS)

Low-resolution mass spectra were recorded using an Agilent Technologies 1200 series LC-MS instrument with a 6130 single Quadropole and a dual electrospray and atmospheric chemical ionisation source. LC traces were recorded at a wavelength of 254 nm using an Agilent poroshell 120, EC-C18 2.7 µm, 4.6×100 mm column at 1 mL per minute. Method A was used for chromatographic separation, using solvent A (water with 5 mM ammonium acetate), and solvent B (MeCN with 5 mM ammonium acetate). Total lunch time: 18 min.

Table S3: LR-MS method.

**Time (min.sec)**	**Flow Rate (mL/min)**	**% Solvent A**	**% Solvent B**
**0 s**	1	95	05
**1.48**	1	95	05
**8.50**	1	00	100
**13.50**	1	00	100
**16.50**	1	95	05
**18.00**	1	95	05

High-Resolution Mass Spec (HR-MS)

This was carried out at NMSF at Swansea University. Samples were solvated in MeCN, then diluted into either MeOH (salts) or MeOH + 30 mM ammonium acetate (neutrals) (to promote protonation and ammonium adduct formation rather than sodiation) for positive ion or diluted into either MeOH (salts) or MeOH + diethylamine (neutrals) for negative ion analysis. The Advion Triversa NanoMate (nano-electrospray) was used to deliver the appropriately diluted samples at a flow of approximately 0.25µL/min. This inlet is used with a 96-well plate, corresponding transfer tips and 400-nozzle spray-chip. Applied nanoelectrospray settings include: spray voltage 1.4 kV, gas pressure 0.4 psi, transfer capillary temperature 200 ^{o}C and transfer capillary voltage 30 V. Mass spectrometric detection was via a Thermo Scientific LTQ Orbitrap XL in positive/negative ionization modes. The instrument is externally calibrated each day using an in-house solution of Caffeine, MRFA (Met-Arg-Phe-Ala), and Ultramark 1621 for positive ion and this solution with added Sodium Dodecyl Sulfate and Sodium Taurocholate for negative ion. Internal calibration is accomplished via 'lockmass' of known background ions. Spectra were recorded at Resolution 100,000 (FWHM) over the m/z range 150 to 2000 Da with a mass accuracy of <3 ppm RMS.

Synthesis of S-MGB-528 and S-MGB-529

<u>Head Group</u>

The phosphonate ester methyl 6-((diethoxyphosphoryl)methyl)nicotinate **(2)** was synthesised via the Arbuzov reaction of methyl 6-(bromomethyl)nicotinate **(1)** (1.0 g) in Triethyl Phosphite (10 mL), as detailed in **Scheme 1.** A subsequent Horner–Wadsworth–Emmons reaction with methyl 6-((diethoxyphosphoryl) methyl)nicotinate **(2)** (600 mg, 2.09 mmol, 1 Eq) and 4-ethynylbenzaldehyde **(3)** (272 mg, 2.09 mmol, 1 Eq), followed by hydrolysis with LiOH (76 mg,

3.18 mmol, 5 Eq) afforded the head group acid **(E)-6-(4-ethynylstyryl)nicotinic acid (4)** (149 mg, 0.60 mmol, 30 %).

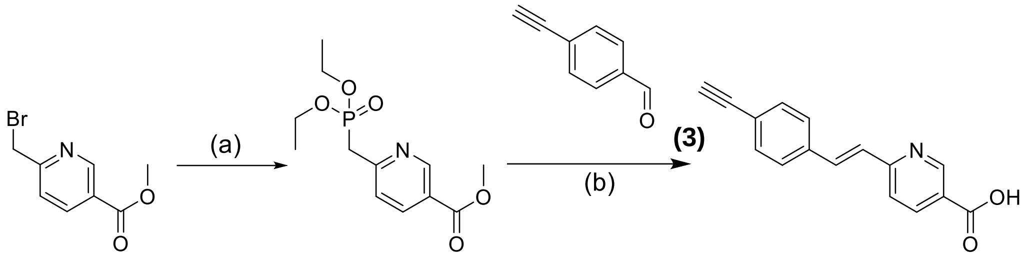

(1) **(2)** **(4)**

Scheme (1): Synthetic route utilised to generate head group moiety (**4**). *Reagents and conditions (a) $P(OEt)_3$, 160 ºC, overnight; (b) (i) NaH, THF (dry), 4-ethynylbenzaldehyde (**3**), 0 ºC – room temperature; (ii) LiOH, H_2O, THF (dry), room temperature, overnight.*

<u>Final S-MGB</u>

Compounds (1-methyl-4-(1-methyl-4-nitro-1H-pyrrole-2-carboxamido)-N-(2-morpholinoethyl)-1H-pyrrole-2-carboxamide) **(5)** and N-(3-amino-3-iminopropyl)-1-methyl-4-(1-methyl-4-nitro-1H-pyrrole-2-carboxamido)-1H-pyrrole-2-carboxamide **(6)** were synthesised via the synthetic methods previously detailed.[1,2] Hydrogenation of compounds 1-methyl-4-(1-methyl-4-nitro-1H-pyrrole-2-carboxamido)-N-(2-morpholinoethyl)-1H-pyrrole-2-carboxamide **(5)** and N-(3-amino-3-iminopropyl)-1-methyl-4-(1-methyl-4-nitro-1H-pyrrole-2-carboxamido)-1H-pyrrole-2-carboxamide **(6)** to afford compounds 4-amino-1-methyl-N-(1-methyl-5-((2-morpholinoethyl)carbamoyl)-1H-pyrrol-3-yl)-1H-pyrrole-2-carboxamide **(7)** and 4-amino-N-(5-((3-amino-3-iminopropyl)carbamoyl)-1-methyl-1H-pyrrol-3-yl)-1-methyl-1H-pyrrole-2-carboxamide **(8)** was achieved employing Pd/C (10% w/w) in MeOH in the presence of H_2 for 4 hours. Amine intermediates 4-amino-1-methyl-N-(1-methyl-5-((2-morpholinoethyl)carbamoyl)-1H-pyrrol-3-yl)-1H-pyrrole-2-carboxamide **(7)** and 4-amino-N-(5-((3-amino-3-iminopropyl)carbamoyl)-1-methyl-1H-pyrrol-3-yl)-1-methyl-1H-pyrrole-2-carboxamide **(8)** were taken forward directly without further purification.

Amide coupling of the generated 4-amino-1-methyl-N-(1-methyl-5-((2-morpholinoethyl)carbamoyl)-1H-pyrrol-3-yl)-1H-pyrrole-2-carboxamide **(7)** (97 mg, 0.24 mmol, 1.2 Eq), with the head group acid (E)-6-(4-ethynylstyryl)nicotinic acid **(9)** (30 mg, 0.12 mmol, 1.0 Eq) in the presence of HATU (2.0 eq) and DIPEA (1.5 eq) in DMF, was carried out as per the synthetic route detailed in **Scheme 2.** The reaction was purified by HPLC purification **(Run Method 1)** and fractions containing the desired compound were subject to freeze drying to afford the title compound **(E)-6-(4-ethynylstyryl)-N-(1-methyl-5-((1-methyl-5-((2-morpholinoethyl) carbamoyl)-1H-pyrrol-3-yl)carbamoyl)-1H-pyrrol-3-yl)nicotinamide (10)** (18 mg, 0.03 mmol, 24 %), as an orange solid.

(E)-N-(5-((5-((3-amino-3-iminopropyl)carbamoyl)-1-methyl-1H-pyrrol-3-yl)carbamoyl)-1-methyl-1H-pyrrol-3-yl)-6-(4-ethynylstyryl)nicotinamide, **(11)** (10 mg, 0.01 mmol, 7.4 %)**,** was generated via amide coupling of 4-amino-N-(5-((3-amino-3-iminopropyl)carbamoyl)-1-methyl-1H-pyrrol-3-yl)-1-methyl-1H-pyrrole-2-carboxamide **(8)** (95 mg, 0.24 mmol, 1.2 Eq) and (E)-6-(4-ethynylstyryl)nicotinic acid, **(9)** (30 mg, 0.12 mmol, 1.0 Eq) in the presence of HBTU (2.0 eq) in DMF, as detailed in **Scheme 2.** The final compound was purified by HPLC purification **(Run Method 2)**, and the appropriate fractions were subject to freeze drying to obtain the title compound as an orange solid.

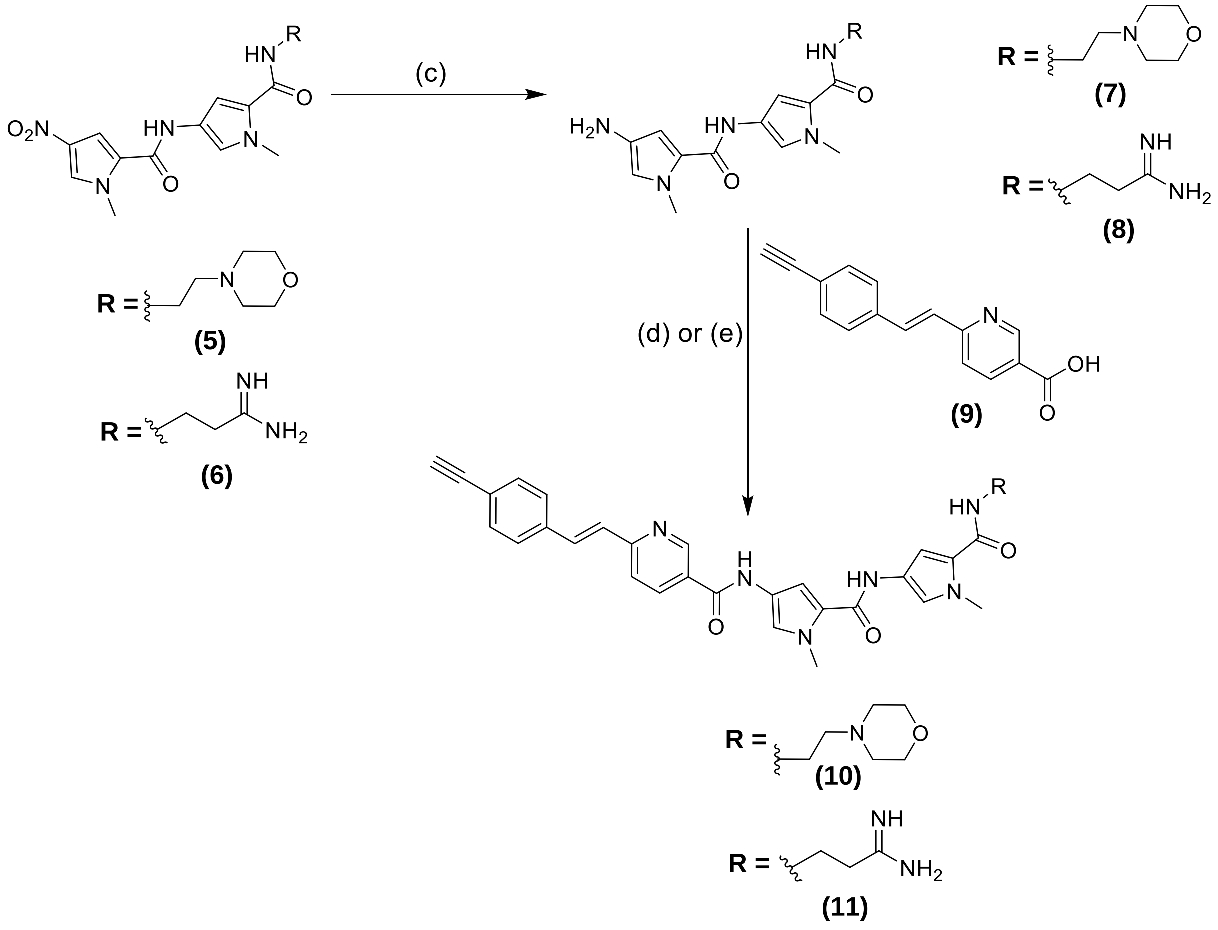

Scheme (2): Synthetic route utilised to generate final S-MGB compounds **(10)** and **(11)**. *Reagents and conditions (c) Pd/C, H_2, MeOH, room temperature, 4 Hr; (d) R = $C_7H_{15}NO$* ***(7)****, HATU, DIEPA, DMF, room temperature, overnight; (e) R = $C_4H_{10}N_2$* ***(8),*** *HBTU, DMF, room temperature, overnight.*

<u>Characterisation</u>

6-(4-ethynylstyryl)nicotinic acid, (9)

^{1}H NMR (500 MHz, DMSO-*d*$_6$) δ (ppm) 9.07 (d, *J* = 2.0 Hz, 1H), 8.28 (dd, *J* = 2.01, 8.03 Hz, 1H), 7.84 (d, *J* = 16.02 Hz, 1H), 7.74 (d, *J* = 8.24 Hz, 2H), 7.70 (d, *J* = 8.10 Hz, 1H), 7.53 (d, *J* = 8.23 Hz, 2H), 7.49 (d, *J* = 16.17, 2H) **^{13}C NMR (126 MHz, DMSO-*d*$_6$)** δ (ppm) 166.33, 158.02, 150.02, 139.30, 136.87, 135.16, 132.66, 131.73, 128.73, 128.16, 127.80, 125.59, 123.16, 122.73, 83.94, 82.80. **LRMS** (ES + APCI) m/z calculated for $C_{16}H_{11}NO_2$ 249.3 $[M]^+$, found 250.1 $[M+H]^+$, at 1.32 min.

(E)-6-(4-ethynylstyryl)-N-(1-methyl-5-((1-methyl-5-((2-morpholinoethyl)carbamoyl)-1H-pyrrol-3-yl)carbamoyl)-1H-pyrrol-3-yl)nicotinamide, (10)

IR (ATR) cm^{-1} 3284.77, 2162.20, 2031.04, 1664.57, 1633.71, 1402.25, 1317.38, 1259.52, 1180.44, 1128.36, 1053.13, 1016.49, 831.32, 798.53, 761.88, 719.45. **^{1}H NMR (500 MHz, DMSO-*d*$_6$)** δ (ppm) 10.51 (s, 1H), 10.00 (s, 1H), 9.51 (m, 1H), 9.12 (d, *J* = 1.83 Hz, 1H), 8.30 (dd, *J* = 2.09, 8.1 Hz, 1H), 8.24 (t, *J* = 5.04 Hz, 1H), 7.81 (d, *J* = 16.02 Hz, 1H), 7.74 (d, *J* = 8.22 Hz, 2H), 7.72 (d, *J* = 7.92 Hz, 1H), 7.54 (d, *J* = 8.24 Hz, 2H), 7.48 (d, *J* = 16.02 Hz, H), 7.36 (d, *J* = 1.78 Hz, 1H), 7.22 (d, *J* = 1.83 Hz, 1H), 7.13 (d, *J* = 1.83 Hz, 1H), 7.02 (d, *J* = 1.38 Hz, 1H), 4.31 (s, 1H), 4.03 (d, *J* = 11.75 Hz, 2H), 3.90 (s, 3H), 3.85 (s, 3H), 3.70 – 3.63 (m,

6H), 3.30 (m, 2H), 3.15 (m, 2H). **LRMS** (ES + APCI) m/z calculated for $C_{34}H_{35}N_7O_4$ 605.7 $[M]^+$, found 606.2 $[M+H]^+$, at 6.92 min. **HRMS** (FTMS + q NSI) m/z calculated for $C_{34}H_{35}N_7O_4$ 606.2823 $[M+H]^+$, found 606.2819 $[M+H]^+$.

(E)-N-(5-((5-((3-amino-3-iminopropyl)carbamoyl)-1-methyl-1H-pyrrol-3-yl)carbamoyl)- 1-methyl -1H-pyrrol-3-yl)-6-(4-ethynylstyryl)nicotinamide, (11)

IR (ATR) cm^{-1} 3265.49, 3041.74, 2154.49, 1660.71, 1631.78, 1589.34, 1581.63, 1516.05, 1433.11, 1406.11, 1288.45, 1265.30, 1199.72, 1176.58, 1128.36, 1006.84, 966.34, 835.18, 798.53, 777.31, 742.59, 657.73. **^{1}H NMR (500 MHz, DMSO-d_6)** δ 10.50 (s, 1H), 9.97 (s, 1H), 9.12 (d, *J* = 2.12 Hz, 1H), 8.9 (s, 2H), 8.45 (s, 2H), 8.30 (dd, *J* = 2.27, 8.09 Hz) 1H), 8.2 (t, *J* = 5.8 Hz, 1H), 7.82 (d, *J* = 16.32 Hz, 1H), 7.74 (d, *J* = 8.24 Hz, 2H), 7.72 (d, *J* = 7.78 Hz, 1H), 7.51 (d, *J* = 8.24 Hz, 2H), 7.48 (d, *J* = 16.32 Hz, 1H), 7.35 (d, *J* = 1.68 Hz, 1H), 7.19 (d, *J* = 1.68 Hz, 1H), 7.12 (d, *J* = 1.83 Hz, 1H), 6.98 (d, *J* = 1.83 Hz, 1H), 4.13 (s, 1H), 3.51 (m, 4H), 3.89 (s, 3H), 3.86 (s, 3H). **LRMS** (ES + APCI) m/z calculated for $C_{31}H_{30}N_8O_3$ 562.6 $[M]^+$, found 563.3 $[M+H]^+$, at 6.52 min. **HRMS** (FTMS + q NSI) m/z calculated for $C_{31}H_{30}N_8O_3$ 563.2510 $[M+H]^+$, found 563.2514 $[M+H]^+$.

TM Data: S-MGB-528 and S-MGB-529

Melting temperatures of gDNA and gDNA:S-MGB complexes calculated from fitted Boltzmann distributions using OriginPro 2021. All values are an average for n=3 experimental repeats.

Table S4: S-MGB-528 complex experiment results X_0 values.

RUN ID	gDNA X_0	gDNA:S-MGB-528 Complex X_0	Difference (ºC)
**1A**	69.35	77.75	8.40
**1B**	69.76	77.17	7.41
**2B**	70.45	77.62	7.17

Table S5: S-MGB-529 complex experiment results X_0 values.

RUN ID	gDNA X_0	gDNA:S-MGB-529 Complex X_0	Difference (ºC)
**1A**	68.82	> 90	> 20
**2A**	69.94	> 90	> 20
**2B**	69.42	> 90	> 20

Table S6: gDNA and gDNA:S-MGB-528 complex graph model details.

S-MGB-528	Model	Equation	A1	A2	X_0	dx	Reduced Chi-Sqr	R-Square (COD)	Adj. R-Square
**gDNA 1A**	Boltzmann	y = A2 + (A1-A2)/(1 + exp((x-x0)/dx))	-0.00352 ± 0.00291	0.97694 ± 0.00278	69.35057 ± 0.05659	2.88601 ± 0.04982	2.33604E-4	0.99879	0.99875
**gDNA 1B**	Boltzmann	y = A2 + (A1-A2)/(1 + exp((x-x0)/dx))	0.00729 ± 0.00695	0.87002 ± 0.00683	69.75552 ± 0.16092	3.15403 ± 0.14264	0.00127	0.99131	0.99104
**COM 1A**	Boltzmann	y = A2 + (A1-A2)/(1 + exp((x-x0)/dx))	0.06773 ± 0.0033	0.95745 ± 0.00508	77.75401 ± 0.06968	1.69185 ± 0.06043	5.81823E-4	0.99635	0.99624
**COM 1B**	Boltzmann	y = A2 + (A1-A2)/(1 + exp((x-x0)/dx))	0.05375 ± 0.00343	0.98539 ± 0.00507	77.17142 ± 0.06687	1.63059 ± 0.05802	6.20298E-4	0.99654	0.99643
**gDNA 2B**	Boltzmann	y = A2 + (A1-A2)/(1 + exp((x-x0)/dx))	0.02606 ± 0.00706	1.03671 ± 0.00726	70.45076 ± 0.1312	2.53045 ± 0.11473	0.00162	0.99244	0.9922
**COM 2B**	Boltzmann	y = A2 + (A1-A2)/(1 + exp((x-x0)/dx))	0.08403 ± 0.00349	0.93256 ± 0.00536	77.6196 ± 0.0787	1.76483 ± 0.06826	6.39179E-4	0.99559	0.99545

Table S7: gDNA and gDNA:S-MGB-529 complex graph model details.

S-MGB-529	Model	Equation	A1	A2	X_0	dx	Reduced Chi-Sqr	R-Square (COD)	Adj. R-Square
**gDNA 1A**	Boltzmann	y = A2 + (A1-A2)/(1 + exp((x-x0)/dx))	0.75814 ± 0.0027	1.00312 ± 0.00252	68.81631 ± 0.19179	2.36737 ± 0.16734	2.23101E-4	0.98268	0.98215
**gDNA 2A**	Boltzmann	y = A2 + (A1-A2)/(1 + exp((x-x0)/dx))	-0.00267 ± 0.0022	1.02738 ± 0.00207	68.94164 ± 0.03749	2.39363 ± 0.03272	1.48412E-4	0.99934	0.99932
**COM 2A**	Boltzmann	y = A2 + (A1-A2)/(1 + exp((x-x0)/dx))	0.13427 ± 0.00427	5.31945 ± 4.67367	103.52464 ± 6.43112	5.34403 ± 0.49111	8.48537E-4	0.97921	0.97857
**COM 1A**	Boltzmann	y = A2 + (A1-A2)/(1 + exp((x-x0)/dx))	0.69175 ± 0.00392	1.08201 ± 0.11426	92.7721 ± 1.23787	1.78762 ± 0.51388	0.00122	0.76754	0.76035
**gDNA 2B**	Boltzmann	y = A2 + (A1-A2)/(1 + exp((x-x0)/dx))	-0.04262 ± 0.00291	0.96818 ± 0.00281	69.42075 ± 0.05225	2.51089 ± 0.04568	2.59758E-4	0.99878	0.99874
**COM 2B**	Boltzmann	y = A2 + (A1-A2)/(1 + exp((x-x0)/dx))	0.16235 ± 0.00673	1.24279 ± 0.20074	92.51052 ± 0.94896	2.22117 ± 0.37213	0.00338	0.90706	0.90418

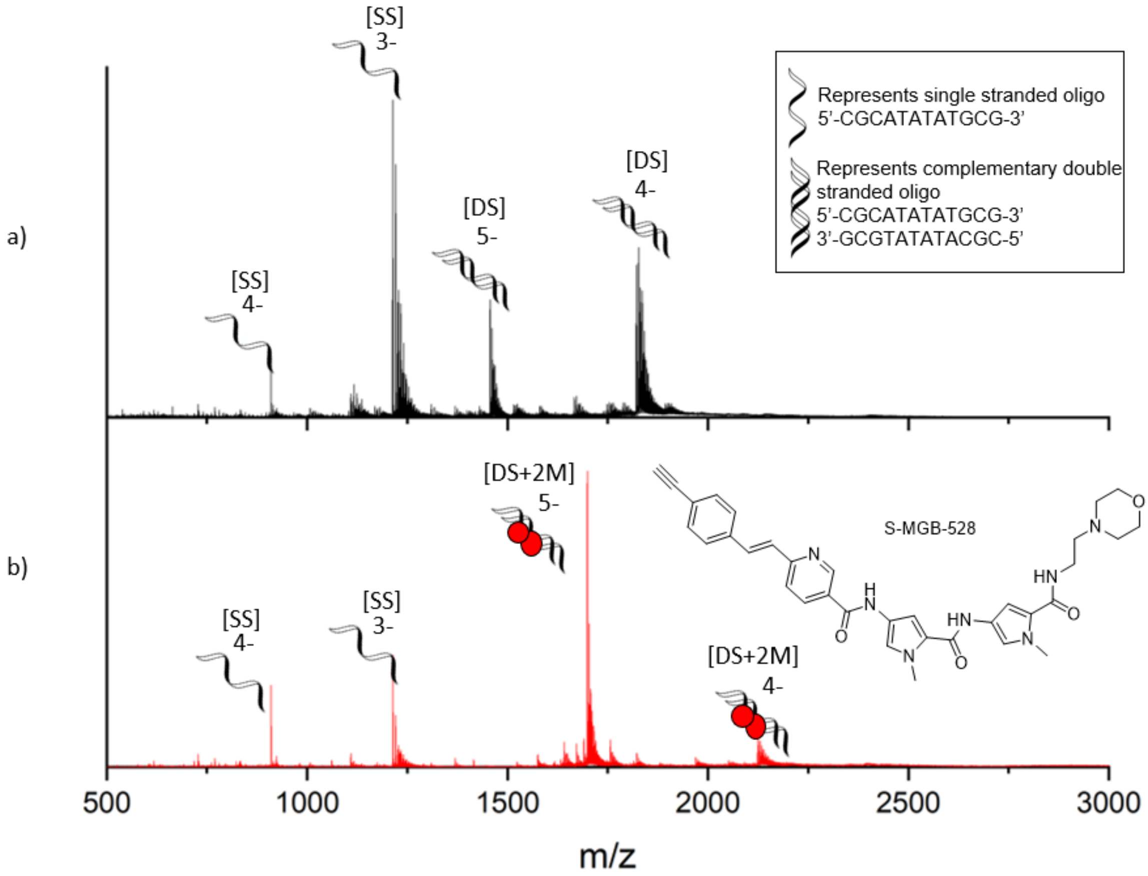

Figure S1: Characterisation of S-MGB-528 binding to double stranded DNA as a dimer by native mass spectrometry (nMS). nMS of DNA sequence 5'-CGCATATATGCG-3' (9 μM DNA, 100 μM KCl, 1% DMSO) sprayed from ammonium acetate (150 mM, pH 7) in the absence **(a)** and presence **(b)** of 100 μM S-MGB-528. **(a)** Single stranded DNA (denoted [SS]) are present in charge states 4- and 3-, and double stranded DNA (denoted [DS]) are present in charge states 5-$^{-}$ and 4-. **(b)** [SS] is present in charge state 3- and 4-. Each [DS] molecule is seen to bind 2xS-MGB molecules (denoted [DS+2M]) and is present in charge states 5-and 4-.

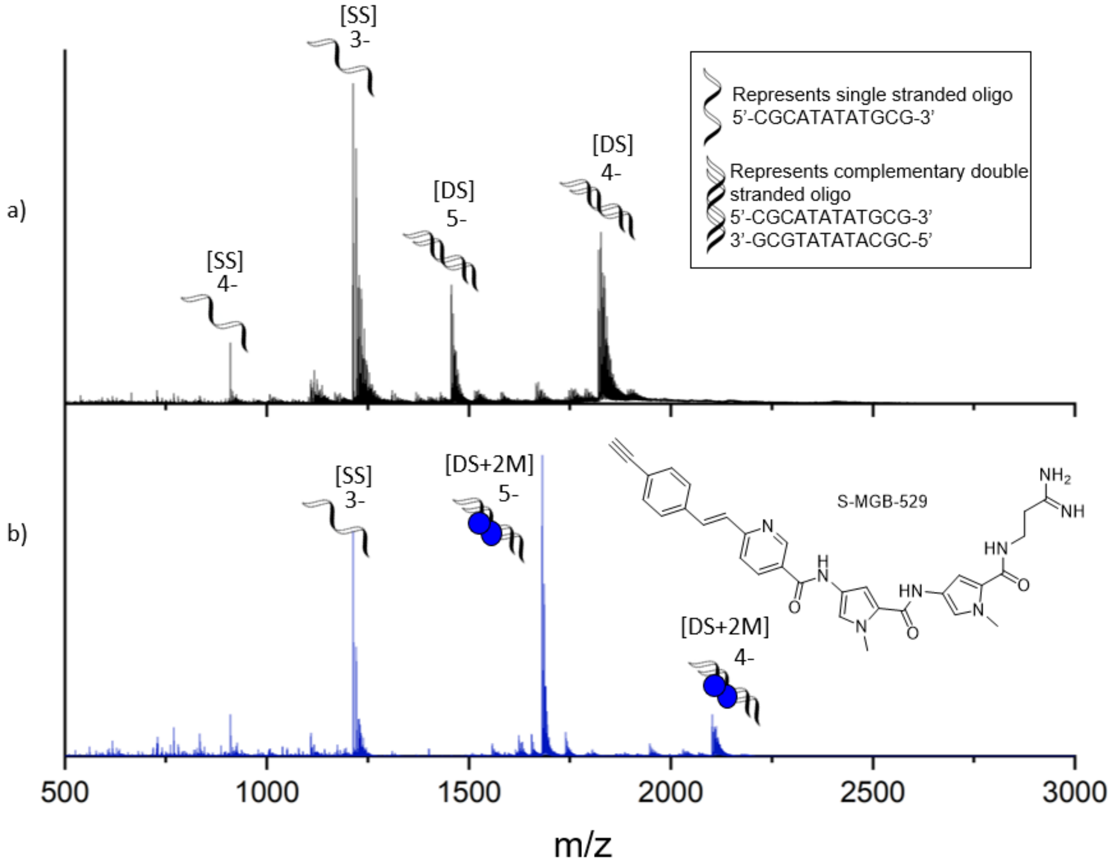

Figure S2: Characterisation of S-MGB-529 binding to double stranded DNA as a dimer by nMS. nESI-MS of DNA sequence 5'-CGCATATATGCG-3' (9 μM DNA, 100 μM KCl, 1% DMSO) sprayed from ammonium acetate (150 mM, pH 7) in the absence **(a)** and presence **(b)** of 100 μM s-MGB. **(a)** Single stranded DNA (denoted [SS]) are present in charge states 4- and 3-, and double stranded DNA (denoted [DS]) are present in charge states 5-$^{-}$ and 4-. **(b)** [SS] is present in charge state 3-. Each [DS] molecule is seen to bind 2xS-MGB molecules (denoted [DS+2M]) and is present in charge states 5-and 4-.

Table S8: Calculated and measured masses for each species observed in **Figure S1** for DNA sequence 5'-CGCATATATGCG-3' S-MGB-528.

Species	m/z value	Calculated mass of neutral species (Da)
**Single Stranded [SS]**	3- : 1214.1<br>4- : 910.3	(1214.1*3) + 3 = 3645.3<br>(910.3*4) + 4 = 3645.2
**Double Stranded [DS]**	4- : 1821.7<br>5- : 1457.2	(1821.7*4) + 4 = 7290.8<br>(1457.2*5) + 5 = 7291.0
Double Stranded + 2 x S-MGB-528 [DS+2M]	4- : 2124.5<br>5- : 1699.4	(2124.5*4) + 4 = 8502.0<br>(1699.4*5) +5 = 8502.0

Table S9: Calculated and measured masses for each species observed in **Figure S2** for DNA sequence 5'-CGCATATATGCG-3' S-MGB-529.

Species	m/z value	Calculated mass of neutral species (Da)
**Single Stranded [SS]**	3- : 1214.1<br>4- : 910.3	(1214.1*3) + 3 = 3645.3<br>(910.3*4) + 4 = 3645.2
**Double Stranded [DS]**	4- : 1821.7<br>5- : 1457.2	(1821.7*4) + 4 = 7290.8<br>(1457.2*5) + 5 = 7291.0
Double Stranded + 2 x S-MGB-529 [DS+2M]	4- : 2102.7<br>5- : 1682.0	(2102.7*4) + 4 = 8414.8<br>(1682.0*5) + 5 = 8415.0

Mass Spec Discussion

Native mass spectrometry experiments were used to demonstrate that both S-MGB-528 and S-MGB-529 bind to double stranded DNA, using a short DNA oligo with an AT rich binding site (5'-CGCATATATGCG-3'). nESI-MS was also used to confirm that both S-MGB-528 and S-MGB-529 bind to double stranded DNA as dimer species, as expected of molecules of this type.

Analysis of the DNA sequence 5'-CGCATATATGCG-3' by nESI-MS revealed the presence of single stranded DNA [SS] in charge states 4- and 3-, and double stranded DNA [DS] in charge states 5- and 4- **(Figures S1 and S2).** nESI-MS of DNA sequence 5'-CGCATATATGCG-3' in the presence of S-MGBs revealed that both S-MGBs bound to double stranded DNA as a dimer species [DS+2M] in charge states 5- and 4- **(Figure S1 and S2)**. No formation of S-MGB bound to double stranded DNA as a monomer species [DS+ 1M] was observed by nESI-MS. These observations suggest that the S-MGBs selectively bind to double stranded DNA as a dimer species only. No detection of unbound double stranded DNA [DS] was observed by nESI-MS in the presence of the S-MGBs. Expected and measured masses of each species are provided in **Tables S8** and **S9**.

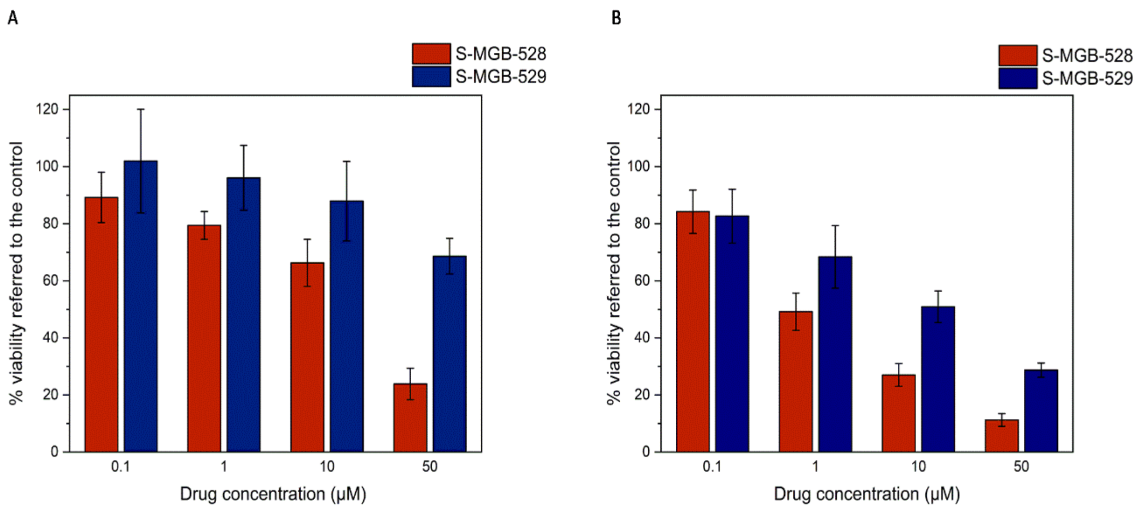

Figure S3: Bar chart showing the activity of either the S-MGB-528 (red) and S-MGB-529 (blue) activity against (A) PNT2 and (B) HeLa cells. Each % is referred to the control (100%). Data represents mean ± SD.

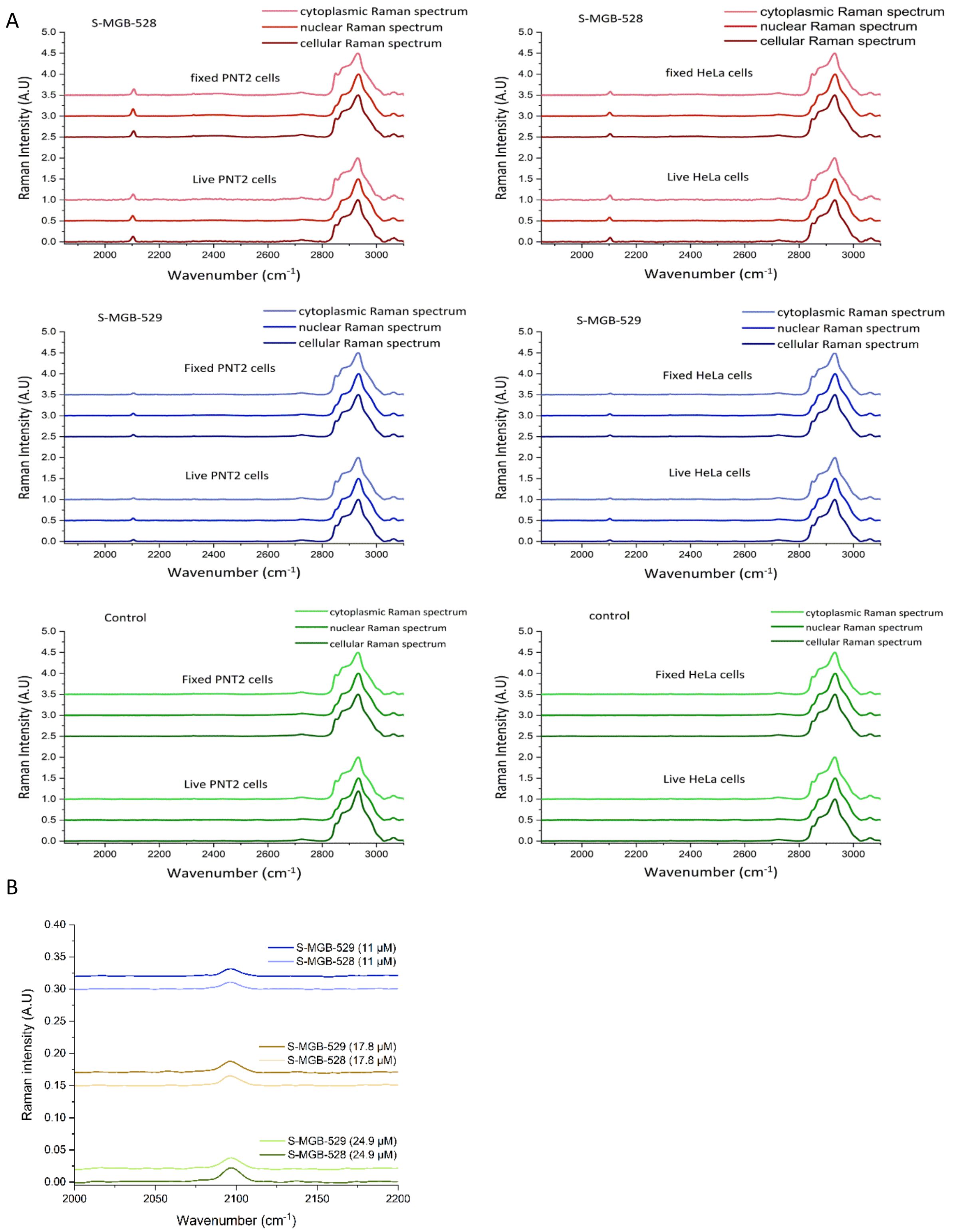

Figure S4: (A) Average Raman spectrum of both live and fixed PNT2 and HeLa cells treated with either S-MGB-528, S-MGB-529 or DMSO (control). Average Raman spectra were determined from the whole cell, nucleus and cytoplasm, which were determined via threshold analysis. A minimum of three replicate images were acquired for each condition. (B) The investigation of the Raman scattering cross section of the alkyne tags of either S-MGB at a concentration of 11 µM, 17.8 µM and 24.9 µM in DMSO. The alkyne Raman scattering was normalised to the Raman peak of DMSO at 2910 cm^{-1}. The Raman spectra of both S-MGB-528 and S-MGB-529 in solution were collected using 532 nm, 0.5 s, 10 mW, 60× immersive objective lens, 1 accumulation. The data suggest negligible differences in the Raman scattering cross section of the molecules.

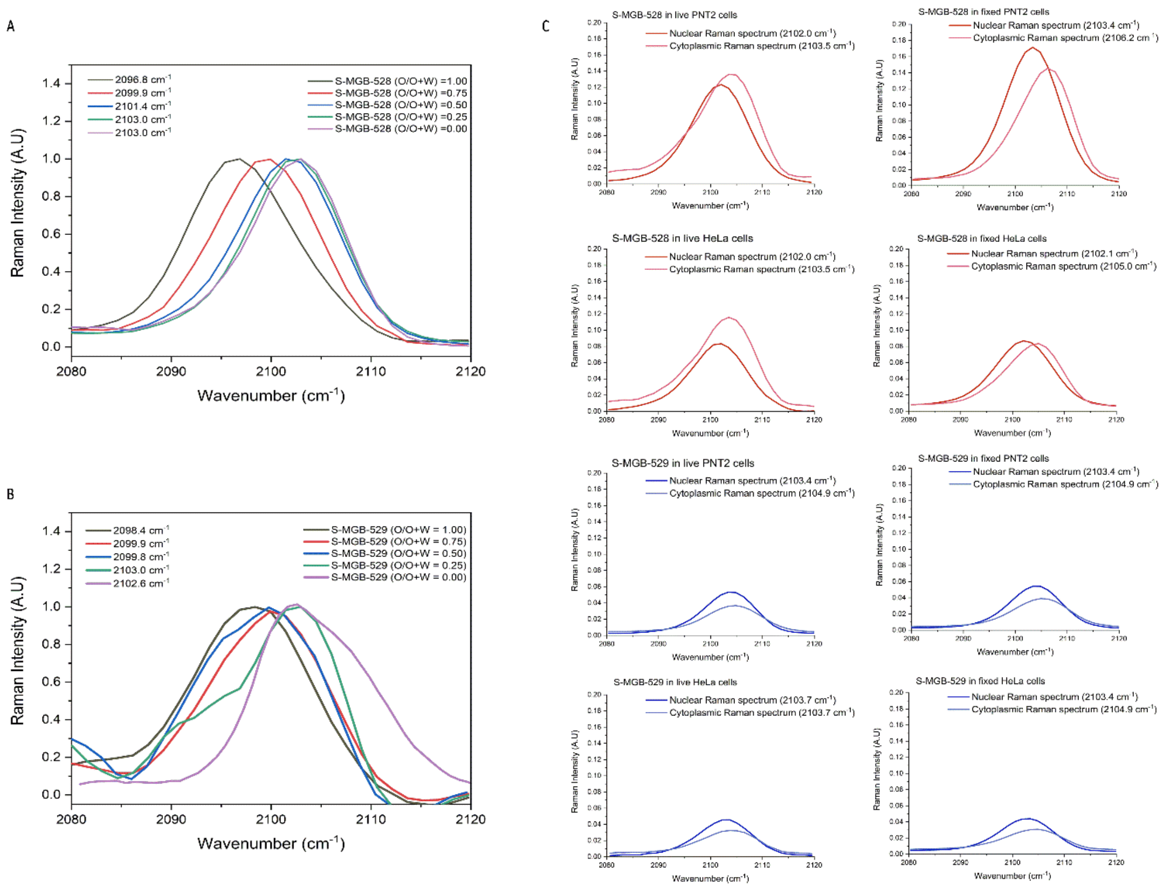

Figure S5: (A)(B) The Raman spectrum of a 20 mM solution of S-MGB-528 and S-MGB-529 moving gradually from an organic to an aqueous phase, using Raman spectroscopy at 532 nm, 0.5 s,10 mW, 60× immersive objective lens, 20 accumulations. (C) A comparison of the nuclear and cytoplasmic average Raman spectrum of either S-MGB-528 or S-MGB-529 in both live and fixed PNT2 and HeLa cells in the nuclear and cytoplasmic region. A red shift of the alkyne tag is detected within the nucleus. The alkyne Raman intensity was normalised to the symmetric C-H stretching (2930 cm^{-1}). A minimum of three replicate images were acquired for each condition.

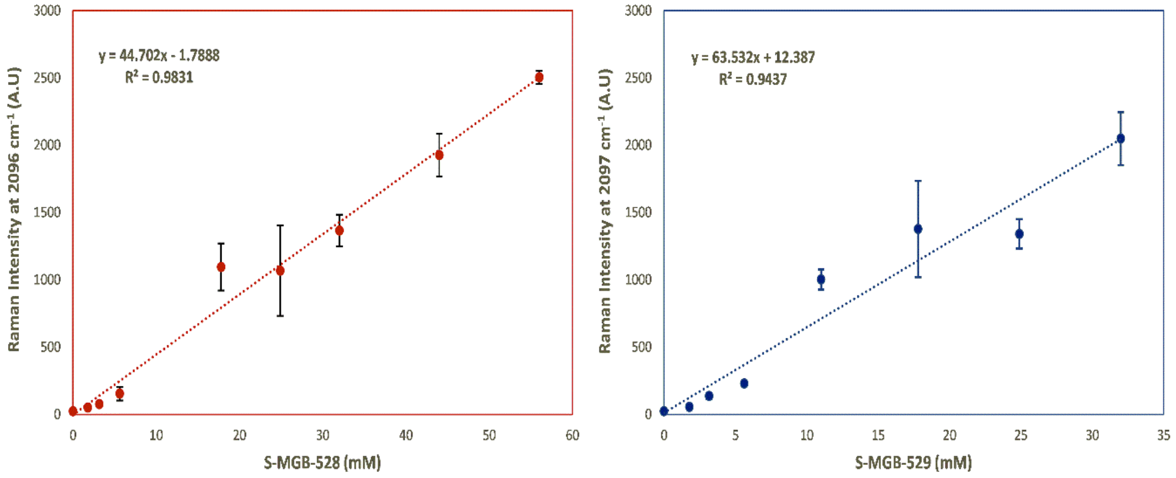

Figure S6: The Raman intensity of the alkyne moiety of S-MGB-528 and S-MGB-529 at 2096 and 2097 cm^{-1} in DMSO solution, respectively. The Raman spectra of both S-MGB-528 and S-MGB-529 in solution were collected using 532 nm, 0.5 s, 10 mW, 60× immersive objective lens, 1 accumulation. The alkyne scattering of S-MGB-529 over the concentration of 32 mM loses its linear relationship with the drug concentration; hence, the different drug concentration ranges used for each MGB. Data represents mean ± SD

Table S10: Estimated concentration of S-MGB-528 and S-MGB-529 in live PNT2 and HeLa cells with the relative enrichment ratio taking into account the extracellular drug concentration. Data represents mean ± SD. A minimum of three replicate images were acquired for each condition.

**S-MGB-528**	[mM] in live PNT2 cells	Enrichment ratio	[mM] in live HeLa cells	Enrichment ratio
Whole-cell	(1.29 ± 0.16)	(129 ± 16)	(1.40 ± 0.07)	(140 ± 7)
Nuclear region	(1.29 ± 0.23)	(129 ± 23)	(1.02 ± 0.26)	(102 ± 26)
Cytoplasmic region	(1.32 ± 0.14)	(132 ± 14)	(1.45 ± 0.07)	(145 ± 7)
**S-MGB-529**				
Whole-cell	(0.43 ± 0.31)	(43 ± 31)	(0.13 ± 0.06)	(13 ± 6)
Nuclear region	(0.40 ± 0.28)	(40 ± 28)	(0.27 ± 0.03)	(27 ± 3)
Cytoplasmic region	(0.35 ± 0.31)	(35 ± 31)	(0.9 ± 0.09)	(9 ± 9)
**Cellular(S-MGB-528/S-MGB-529)**	≈3		≈10	
**Nuclear(S-MGB-528/S-MGB-529)**	≈3		≈5	
**Cytoplasmic(S-MGB-528/S-MGB-529)**	≈3		≈14	

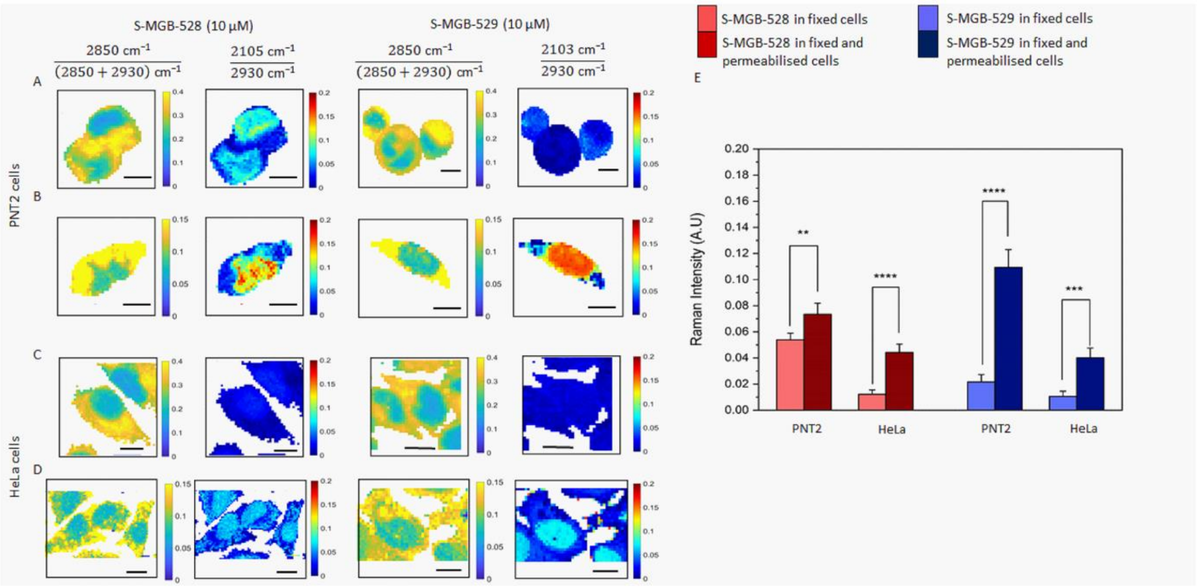

Figure S7: The investigation of the lipid membrane on the S-MGBs uptake. The intracellular uptake of S-MGB-528 and S-MGB-529 in either fixed (A) PNT2 and (C) HeLa cells or fixed and permeabilised (B) PNT2 and (D) HeLa cells. The cells were fixed using a 4% PFA solution in PBS (37°C, 10 mins), incubated with PBS in the presence or absence of TRITON-X-100 (0.1%, 10 mins) and treated with S-MGB-528 and S-MGB-529 (10 µM, 10 mins). (E) Normalised cellular alkyne intensity of S-MGB-528 and S-MGB-529 to the symmetric C-H stretching (2930 cm^{-1}) in either fixed or fixed and permeabilised PNT2 and HeLa cells. Raman data were collected using 532 nm, 0.5 s, 10 mW, 60× immersive objective lens, step size 1 micron in x and y, 1 accumulation. This analysis readily resolves the nuclear (ratio 1 ≈ 0.2 au) and cytoplasmic regions (typically ratio 1 >0.25 au) of the fixed cells and the nuclear (ratio 1 ≤ 0.1 au) and cytoplasmic regions (typically ratio 1 > 0.1 au) of the fixed and permeabilised cells. Data represents mean ± SD (**** $p \leq 0.0001$; *** $p \leq 0.001$; ** $p \leq 0.01$; * $p \leq 0.05$; ns $p > 0.05$, Student's t test). A minimum of three replicate images were acquired for each condition. Scale bar size: 10 micron.

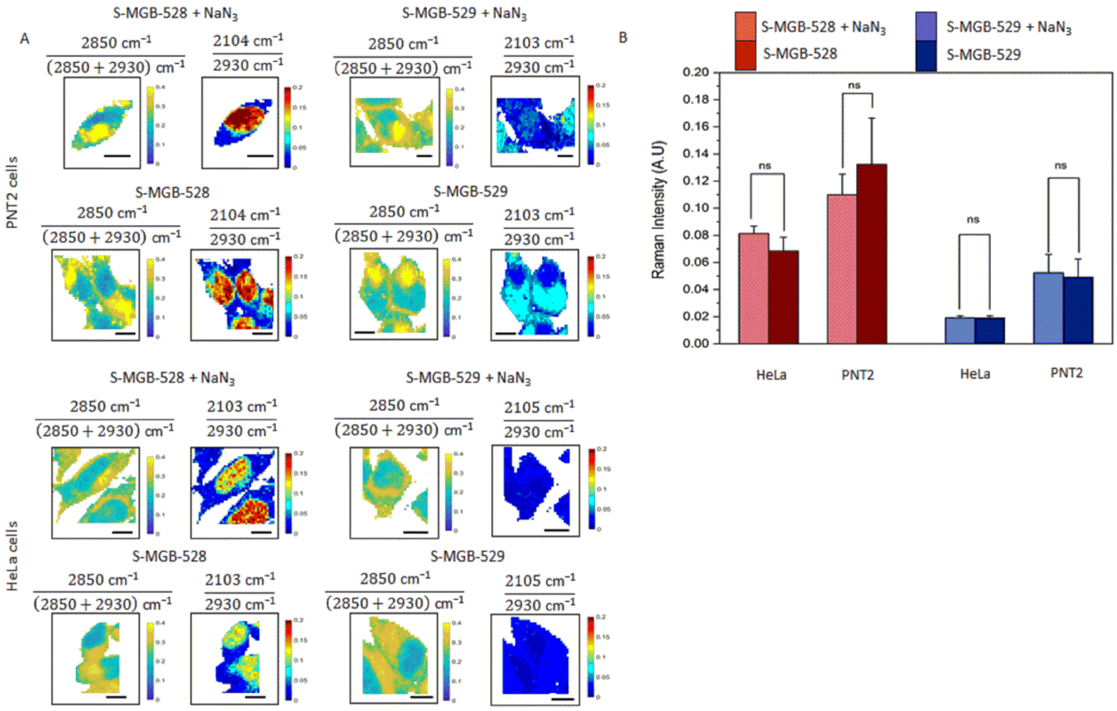

Figure S8: Investigation of active transport as a potential mechanism of MGB uptake. (A) Representative Raman maps of PNT2 and HeLa cells treated with S-MGB-528 or S-MGB-529 in presence or absence of sodium azide. PNT2 and HeLa cells were alternatively preincubated and co-incubated with medium containing sodium azide (5 mM, top row) or without sodium azide (bottom row), respectively. Raman scattering was collected using 532 nm, 0.5 s, 10 mW, 60× immersive objective lens, step size 1 micron in x and y, 1 accumulation. (B) Averaged Raman intensity of the alkyne group at 2104 cm^{-1} (S-MGB-528) and 2103 cm^{-1} (S-MGB-529) normalised to the symmetric C-H stretching (2930 cm^{-1}) in live PNT2 and HeLa cells. PNT2 and HeLa cells were either preincubated and co-incubated with or without sodium azide. Data represents mean ± SD (**** $p \leq 0.0001$; *** $p \leq 0.001$; ** $p \leq 0.01$; * $p \leq 0.05$; ns $p > 0.05$, Student's t test). A minimum of three replicate images were acquired for each condition.

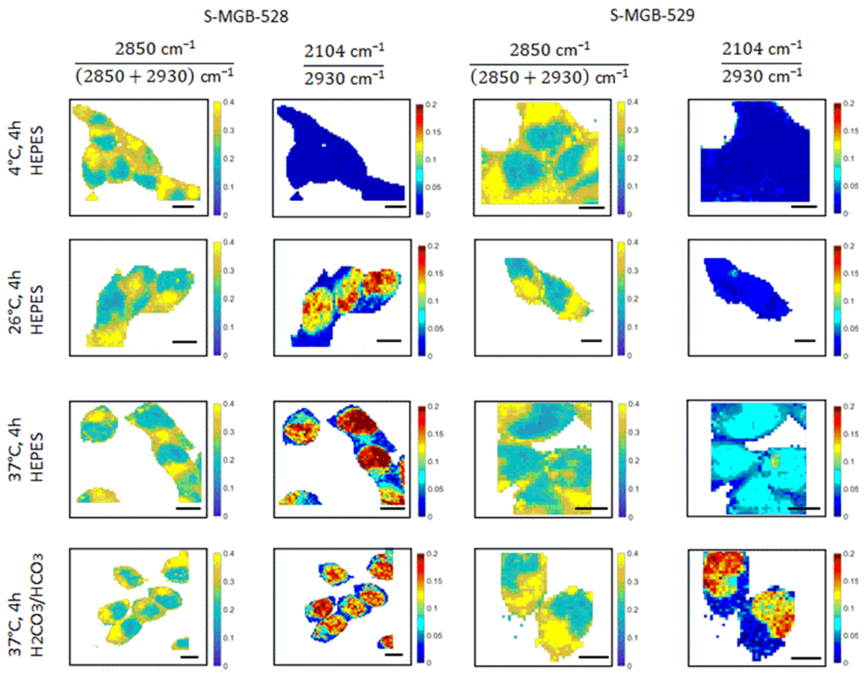

Figure S9: Investigation of the S-MGB uptake temperature dependence in live PNT2 cells. Representative Raman maps of live PNT2 cells after the treatment with S-MGB-528 and S-MGB-529 (10/20 µM, 4h, 4°C/26°C/37°C, in presence of HEPES or bicarbonate buffer). The chemical contrast is shown by the ratio 2850 cm^{-1}/ (2850+2930) cm^{-1} that identifies the localisation of the cells and allows the investigation of the drug's intracellular distribution at sub-cellular resolution. The drug localisation is shown through the normalised alkyne Raman intensity (i.e. 2104 cm^{-1}/2930 cm^{-1}). Raman data were collected using 532 nm, 0.5 s, 10 mW, 60× immersive objective lens, step size 1 micron in x and y, 1 accumulation.

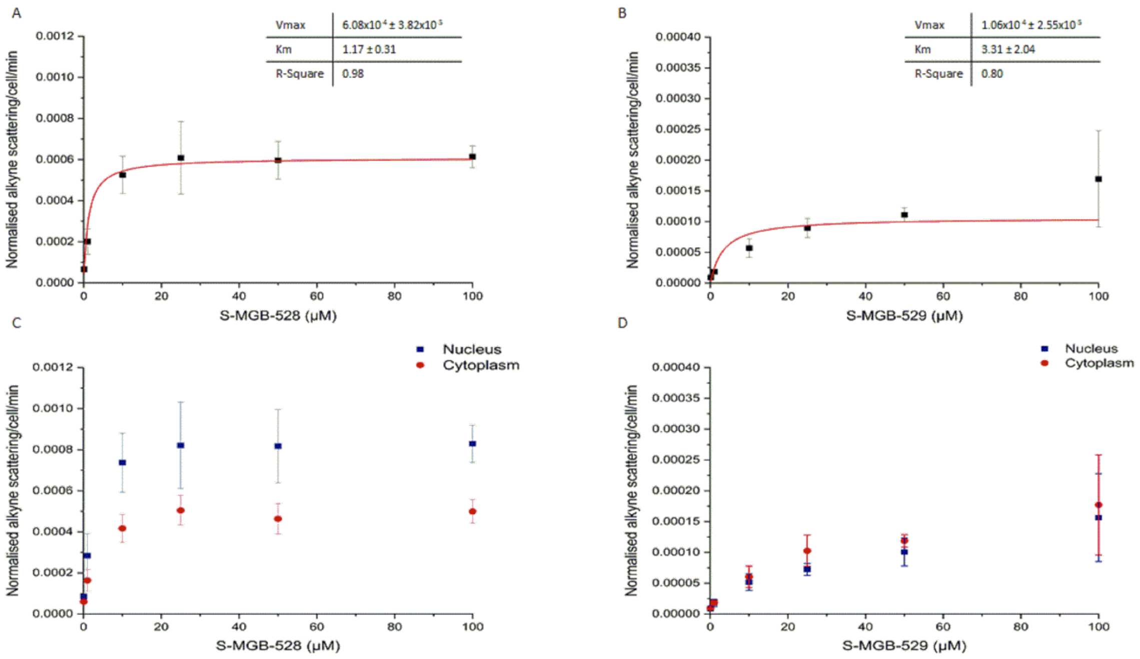

Figure S10: Alkyne scattering of S-MGB-528 and S-MGB-529 in live PNT2 cells at the cellular (top line) and sub-cellular resolution (bottom line). PNT2 cells were incubated with growing concentration of either S-MGB-528 or S-MGB-529 in a humified incubator (4 h). Raman data were collected using 532 nm, 0.5 s, 10 mW, 60× immersive objective lens, step size 1 micron in x and y, 1 accumulation. The alkyne Raman intensity was normalised to the symmetric C-H stretching (2930 cm^{-1}). A minimum of three replicate images were acquired for each condition.

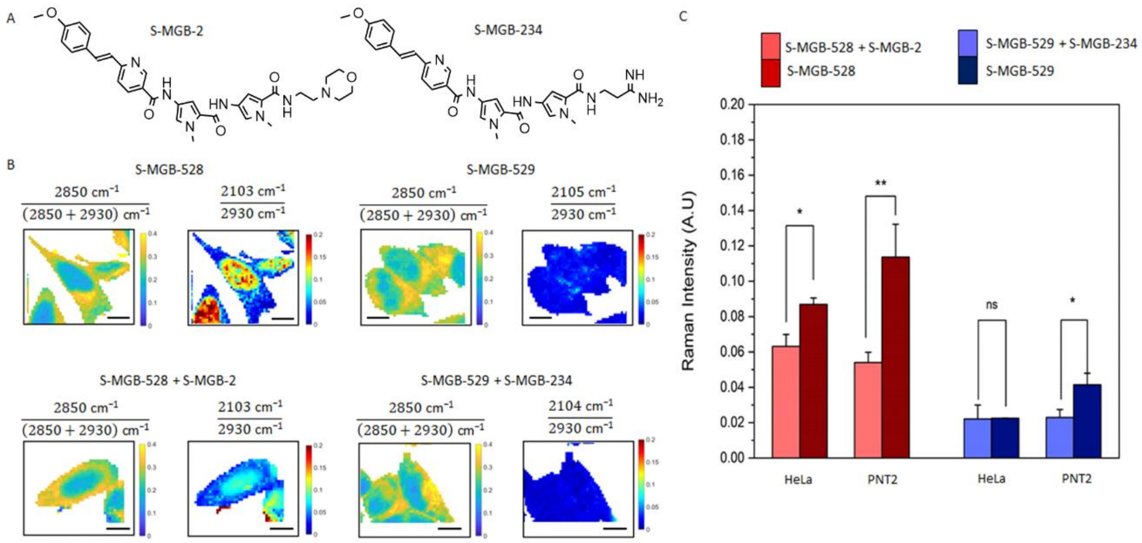

Figure S11: Investigation of the facilitated diffusion as a potential mechanism for MGB uptake using structural analogues. (A) Molecular structure of the MGBs analogues, S-MGB-2 and S-MGB-234. (B) Representative Raman maps of live HeLa cells after the treatment with S-MGB-528 and S-MGB-529 (10/20 µM, 4 h, 37°C) in the presence or absence of the structural analogues, the S-MGB-2 (100 µM) and S-MGB-234 (100 µM), respectively. The chemical contrast is shown by the ratio 2850 cm^{-1} / (2850+2930) cm^{-1} that identifies the localisation of the cells and allows the investigation of the drug's intracellular distribution at sub-cellular resolution. The drug localisation is shown through the normalised alkyne Raman intensity (i.e. 2104 cm^{-1}/2930 cm^{-1}) to the symmetric C-H stretching (2930 cm^{-1}). (C) Normalised alkyne intensity of S-MGB-528 and S-MGB-529 in HeLa and PNT2 cells in the presence or absence of the structural analogues. The Raman data were acquired using 532 nm, 0.5 s, 10 mW, 60× objective immersive lens, step size 1 micron in x and y, 1 accumulation. Data represents mean ± SD (**** $p \le 0.0001$; *** $p \le 0.001$; ** $p \le 0.01$; * $p \le 0.05$; ns $p > 0.05$, Student's t test). A minimum of three replicate images were acquired for each condition.

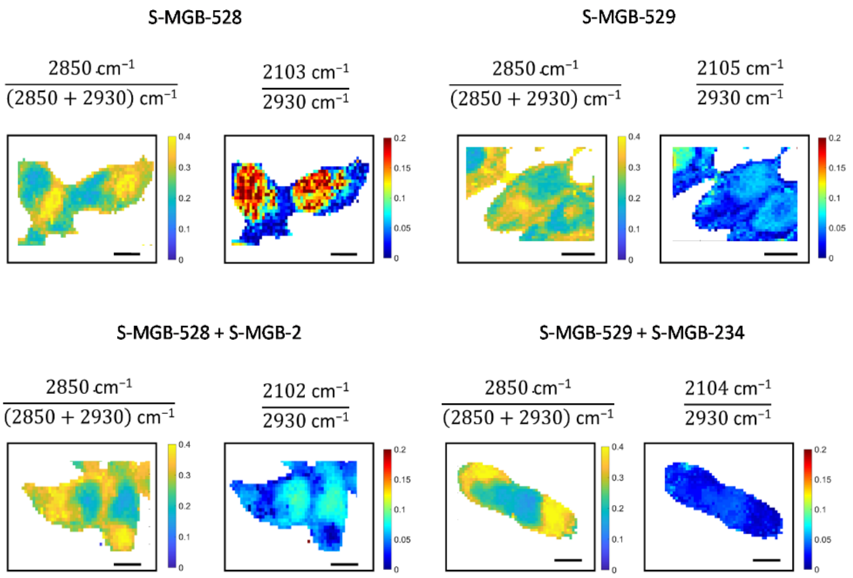

Figure S12: Representative Raman maps of live PNT2 cells after the treatment with S-MGB-528 and S-MGB-529 (10/20 µM, 4 h, 37°C) in the presence or absence of the structural analogues, S-MGB-2 and S-MGB-234, respectively. Raman data were collected using 532 nm, 0.5 s, 10 mW, 60× immersive objective lens, step size 1 micron in x and y, 1 accumulation. The alkyne Raman intensity was normalised to the symmetric C-H stretching (2930 cm^{-1}).

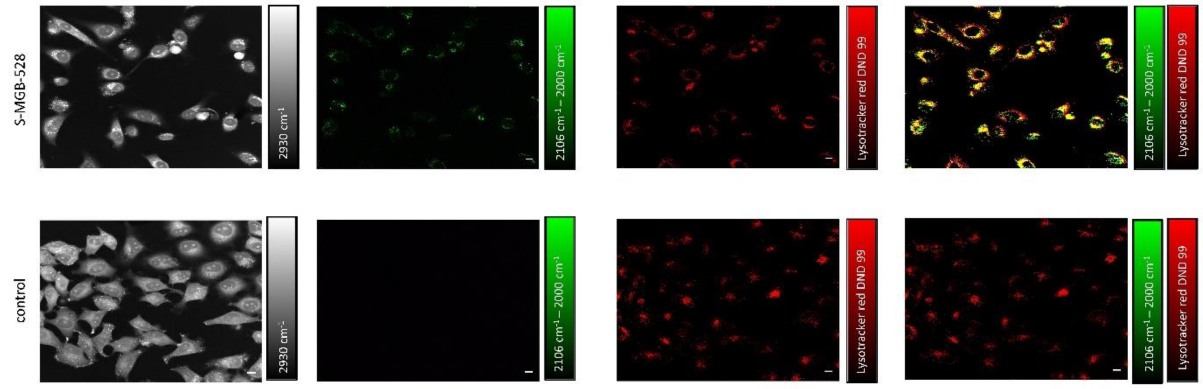

Figure S13: Multimodal imaging of S-MGBs uptake. Representative SRS and fluorescence images of live HeLa cells after the treatment with S-MGB-528 (10 µM) for 6 h and further incubation with lysotracker red DND 99 (50 nM) for 30 mins in incubator. The sample was imaged using SRS microscopy at the following: 2930 cm^{-1} (proteins), 2106 cm^{-1} (alkyne) and 2000 cm^{-1} (off-resonance). The fluorescence of lysotracker was collected using 561 nm, LP2%, 600 V, range 570-650 nm. An image achieved by merging the channels of lysotracker red DND 99 and alkyne (on-off resonance) is also shown highlighting the lysosomal localisation of S-MGB-528. Lookup table: 0-3000 A.U (proteins), 0-1500 A.U (Lysotracker red DND 99 and on-off resonance). Scale bar size: 10 micron.

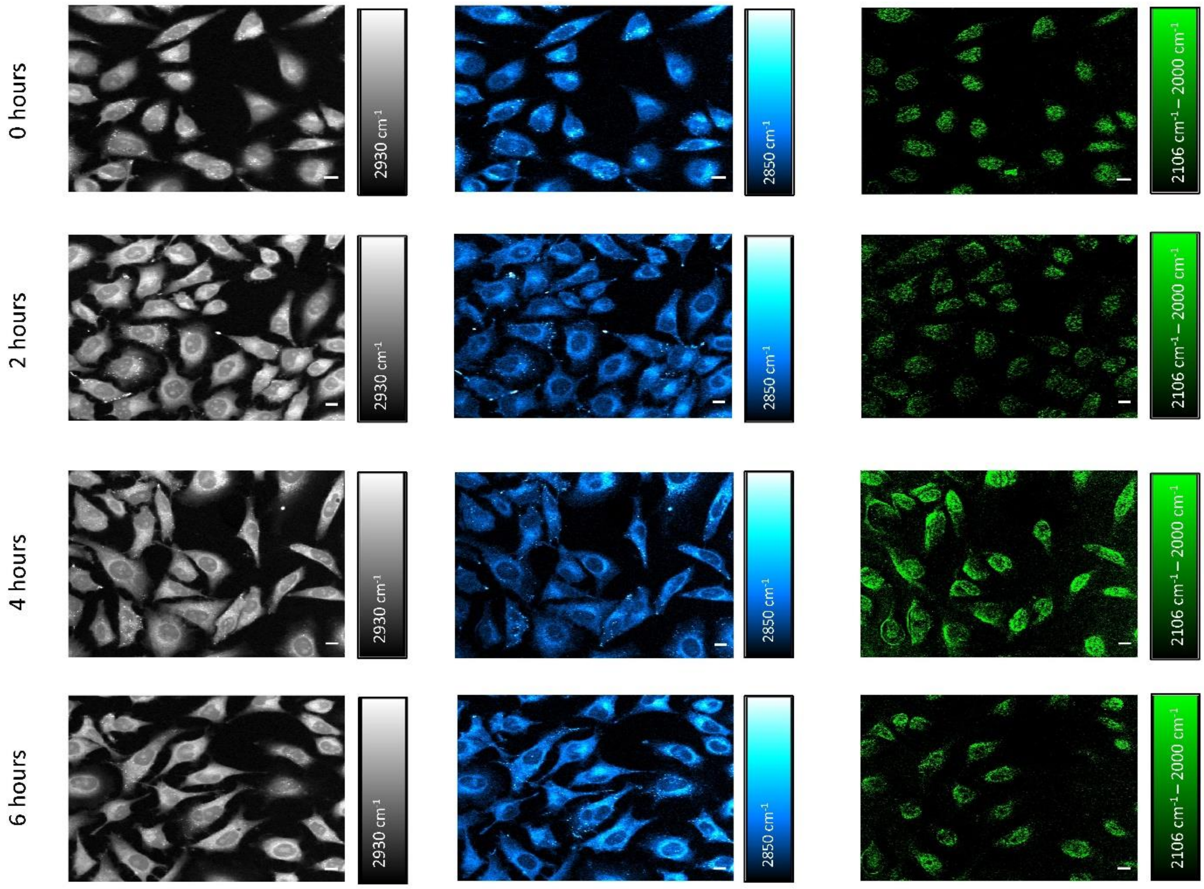

Figure S14: Representative SRS images of live HeLa cells after the treatment with S-MGB-528 (10 µM) for 30 mins and further incubation with fresh DMEM for either 2, 4 or 6 hours in a humified incubator. The sample was imaged using SRS microscopy at the following: 2930 cm^{-1} (proteins), 2850 cm^{-1} (lipids), 2106 cm^{-1} (alkyne) and 2000 cm^{-1} (off-resonance). Lookup table: 0-3000 A.U. (proteins and lipids), 0-1500 A.U. (on-off resonance). Scale bar size: 10 micron.

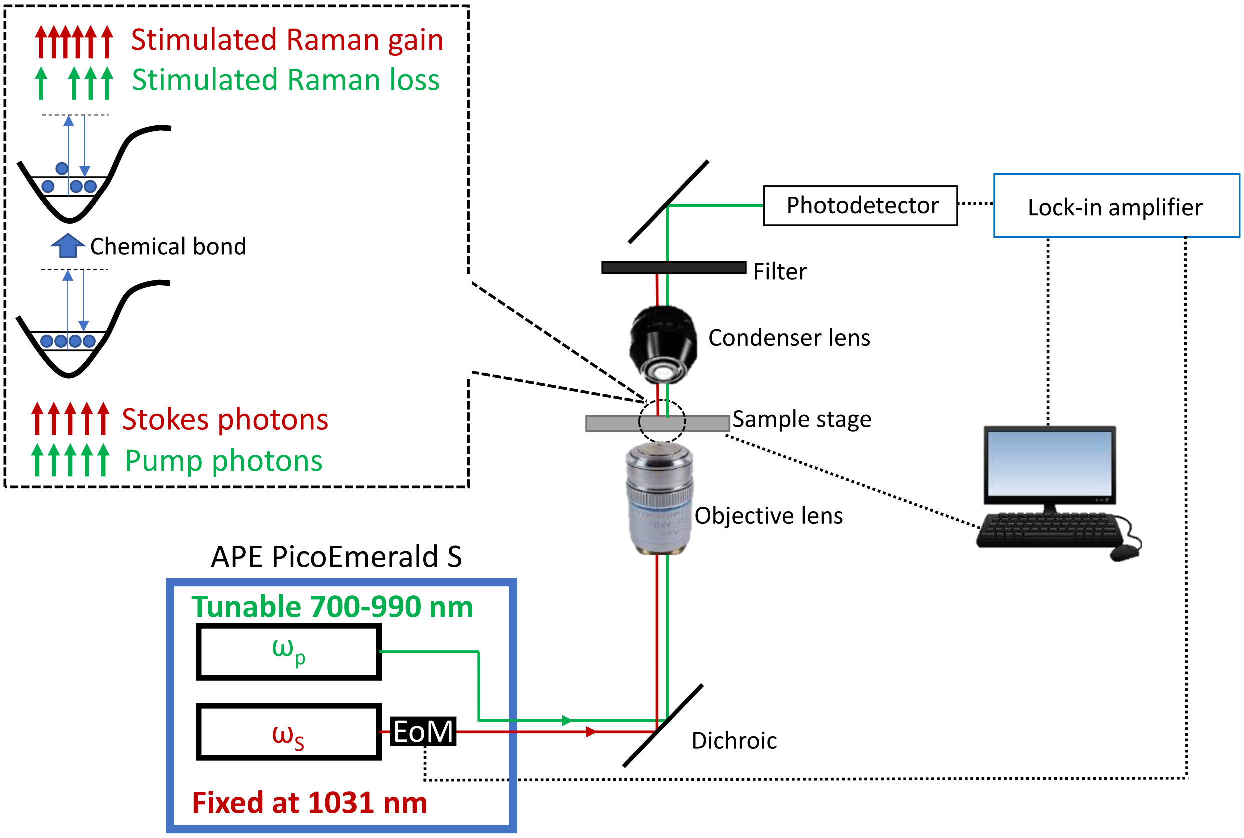

Figure S15 Schematic diagram of the SRS microscope.
